# Shear-free, inhomogeneous turbulence in a stably stratified fluid

**DOI:** 10.1017/jfm.2026.11627

**Published:** 2026-06-16

**Authors:** Ryan Hass, Sanjiva Lele

**Affiliations:** 1 Verification and Analysis (XCP-8), Los Alamos National Laboratory, Los Alamos, NM 87545, USA; 2 Department of Mechanical Engineering, Stanford University, 450 Jane Stanford Way, Stanford, CA 94305, USA; 3 Department of Aeronautics and Astronautics, Stanford University, 450 Jane Stanford Way, Stanford, CA 94305, USA

**Keywords:** stratified turbulence

## Abstract

High-resolution large eddy simulations are conducted of locally forced, shear-free turbulence in the presence of an initially sharp density interface. The simulations are reminiscent of oscillating grid turbulence experiments used to isolate the effect of turbulent diffusion and entrainment from background shear. By simulating such a flow we avoid common challenges of the experiments such as secondary-flow contamination due to sidewall effects and the inevitable interaction of the stratifying agent and forcing region. To address the latter concern, we add a heating term (potential energy sink) to the governing equations in the forcing layer, thereby preventing a heat flux through the source region. This modification sets up a continuous stratification in the mixed layer that is often assumed to be negligible in experiments. Despite this difference, we are able to make meaningful comparisons in terms of the overall entrainment rate, which varies as a power law with a turbulent Richardson number. Two exponents, −2 and −1 , are measured depending on the definition of the Richardson number and entrainment rate used. The definition leading to −1 is consistent with most experiments, and we argue it is the superior choice if one is able to measure the relevant quantities. We also verify the self-similar scaling of turbulence velocity and length scales in the homogeneous fluid and propose ‘inner’ and ‘outer’ scalings for the stratified cases based on a local Froude number. The detailed scaling results are useful for turbulence model validation.

## Graphical abstract

**Figure:**
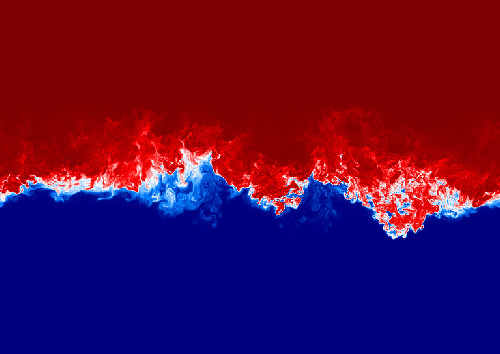

## 1. Introduction

Studies of shear-free turbulent entrainment of a stable density interface were initiated by the pioneering experiments of Rouse & Dodu (1955). Motivated by the analogy to shear-free turbulence injected into the upper ocean via surface wave breaking, and the somewhat ‘simpler’ study of stratified turbulent entrainment absent mean shear, they developed an experimental apparatus that approximated these conditions. A localised layer of turbulence was generated in a tank of water by vertically oscillating a horizontal rigid grid of rectangular bars. The turbulence diffused away from the locus of generation and eventually reached a density interface that was set up prior to the start of the experiment.

They discovered, to their surprise, that rather than establishing a mean density gradient in the turbulent layer, the density interface remained sharp and well defined. The turbulence entrained patches of dense fluid and rapidly broke them down into smaller scales, which then mixed into the turbulent layer. This process was far too fast for a substantial background gradient to form. They found that the rate at which dense fluid was entrained into the upper turbulent layer (i.e. defined in terms of an entrainment velocity $u_{e}$ ) depended on the distance of the grid from the density interface H , the oscillating frequency f , the buoyancy difference across the interface Δb=Δρg/ρ and viscosity ν . Here ρ is the background density, Δρ the density difference across the interface and g is the gravitational acceleration; Δρ is much smaller than ρ , consistent with the Boussinesq approximation (Spiegel & Veronis 1960). The particular functional dependence that they found was

(1.1) $$\begin{array}{r} \frac{u_{e}}{f\;H}∼(\frac{\Delta b}{f^{2}H}{)}^{-\frac{5}{4}}\frac{{f\;H}^{2}}{\nu }. \end{array}$$

Assuming that the turbulence velocity scale near the interface u was proportional to fH and the integral length scale l to H , this expression suggested that

(1.2) $$\begin{array}{r} \frac{u_{e}}{u}∼{Ri}^{-\frac{5}{4}}Re, \end{array}$$

where $Ri=\Delta bl/u^{2}$ and Re=ul/ν are a turbulent Richardson and Reynolds number, respectively.

Parameterising this entrainment rate became the primary focus of subsequent studies. Turner & Kraus (1967) proposed a theoretical model that supports the overall dependence of $u_{e}$ on Ri by supposing that the rate at which kinetic energy is supplied at the grid is proportional to the rate of change of potential energy of the system. Their energy arguments naturally lead to the prediction $u_{e}/u∼{Ri}^{-1}$ . Assuming that viscous effects will become important in regions of strong stratification where turbulence is significantly impeded, a general function $u_{e}/u=f(Ri,Re)$ seems reasonable as was reported in Rouse & Dodu (1955).

To determine possible Prandtl number effects on the entrainment rate, Turner (1968) ran a series of oscillating grid turbulence (OGT) experiments using heat and salt as the stratifying agents in a two-layer system. He found that for sufficiently large Richardson numbers, two distinct power laws developed: ${Ri}^{-1}$ for the heat stratified experiments and ${Ri}^{-3/2}$ for salt. He argued that the ${Ri}^{-1}$ is fundamental based on the energetic arguments of Turner & Kraus (1967) and the ${Ri}^{-3/2}$ dependence is modified due to molecular effects. Based on dimensional arguments he proposed an empirical model that recovers the observed Ri power laws for small and high *Pe,* respectively:

(1.3) $$\begin{array}{r} \frac{u_{e}}{u}={Ri}^{-1}(C+\text{Ri}\text{Pe}{)}^{-\frac{1}{2}}. \end{array}$$

Here C is a constant.

Contrary to the assumption that ${Ri}^{-1}$ should be viewed as fundamental, Linden (1973) proposed a theoretical model that predicts a ${Ri}^{-3/2}$ entrainment rate. The model assumes that a vortex ring represents a prototypical turbulent eddy and that turbulence at the density interface can be approximated as a superposition of many such eddies. The model was validated by projecting successive vortex rings onto a density interface and measuring the resulting entrainment after a certain number of vortex ring events.

Turner (1973) mentioned an unpublished study by C.G.H. Rooth in which heat stratified OGT experiments were able to achieve the ${Ri}^{-3/2}$ dependence by increasing the oscillating frequency (i.e. increasing the Peclet number), validating the notion that the Peclet number enters the functional relationship. (To our knowledge, no published studies have attempted to verify this claim. This is probably due to the difficulty of setting up a thermally stratified two-layer experiment.) Crapper & Linden (1974) firmly established a *Pe* dependence on the dynamics where the interface thickness appeared to be a function of *Pe* only.

Experiments of another kind were also carried out during the same period where the turbulent layer was driven by a surface stress. Whether this was accomplished by sliding a mesh screen over the surface (Kato & Phillips 1969), blowing air over the surface (Wu 1973) or horizontally aligned jets of water (Moore & Long 1971) an entrainment rate $u_{e}/u∼{Ri}^{-1}$ was found. This was perplexing that two different entrainment laws would exist in systems that, by all accounts, had a similar turbulence structure in the mixed layer.

Long (1975) proposed a theory that reconciled the two entrainment rates and concluded that ${Ri}^{-1}$ was fundamental and the ${Ri}^{-3/2}$ dependence reported was due to a weak density gradient in the mixed layer that modified the assumed proportionality u∼fH such that

(1.4) $$\begin{array}{r} \frac{u}{f\;H}∼{Ri}^{-\frac{1}{6}}. \end{array}$$

This slight Richardson number dependence on the velocity scale at the interface u relative to the mechanical stirring rate fH could explain the discrepancy.

In order to say anything definitive, the turbulence would need to be characterised directly and not inferred from the grid geometry and oscillating frequency. Thompson & Turner (1975) were the first to make such measurements, which they did in a homogeneous (unstratified) fluid. They confirmed that the velocity scale is proportional to the oscillating frequency and found that the integral scale grows linearly with distance from the grid, i.e.

(1.5) $$\begin{array}{r} l=\beta z^{\prime }, \end{array}$$

where $z^{\prime }$ is defined as positive moving away from the grid.

The velocity scale was found to possess a power-law decay with distance from the grid that can be anticipated by appealing to the turbulence kinetic energy (TKE) equation, which in the present context, is a balance between transport and dissipation:

(1.6) $$\begin{array}{r} \frac{u}{u_{r}}=(\frac{z^{\prime }}{z_{r}}{)}^{-\frac{B}{3\beta }}. \end{array}$$

Here $u_{r}$ is a reference velocity taken at a reference depth $z_{r}$ .

Equations (1.5) and (1.6) are the primary results of Thompson & Turner (1975). They found that β=0.1 and B/3β≡n=1.5 , but acknowledged that the value of the exponent was very sensitive to the choice of virtual origin. Given this ambiguity, it is unsurprising that a number of values have been measured for the exponent, some of which are reported in table 1.

**Table 1.** Power-law exponents reported in the literature. Table 1 long description.

Study	n
Thompson & Turner (1975)	1.5
Hopfinger & Toly (1976)	1 to 1.25
Hannoun *et al.* (1988)	1
Nokes (1988)	0.8 to 1.5
De Silva & Fernando (1992)	1

In short, the measurements of Thompson & Turner (1975) validate the entrainment rate relationships determined from the grid parameters assuming that turbulence scales in the homogeneous fluid are representative of those responsible for entrainment and mixing at the interface.

To test this latter claim, Hopfinger & Toly (1976) made turbulence measurements in the stratified set-up near the density interface. Their measurements verified the general proportionality $u∼u_{h}∼f$ , where $u_{h}$ is the velocity scale in the homogeneous fluid. These measurements definitively invalidated Long’s suggestion (1.4). Furthermore, Wolanski & Brush Jr (1975) ran a series of OGT experiments using stratifying agents that spanned six orders of magnitude in the diffusivity $\kappa_{i}$ , where ‘ i ’ is for the i th stratifying agent. In the highest Schmidt number ${\text{Sc}}_{i}=\nu /\kappa_{i}$ case they found that $u_{e}/u∼{Ri}^{-4}$ , which, according to Long’s theory, would imply that $u∼f^{3}$ ; Hopfinger & Toly argue that such a scaling is hard to imagine.

Because they found that the *Pe* dependence ceased for high *Pe*, Hopfinger & Toly (1976) proposed an alternative model that recovered the key observations: ${Ri}^{-3/2}$ dependence for sufficiently large Ri and an asymptotic independence of *Pe,*i.e.

(1.7) $$\begin{array}{rl} \frac{u_{e}}{u} & ={Ri}^{-\frac{3}{2}}\left(K_{1}+K_{2}(\frac{Ri}{Pe}{)}^{\frac{1}{2}}\right), \end{array}$$

where $K_{1}$ and $K_{2}$ are constants. The ${Pe}^{-1/2}$ dependence was conjecture and not verified in their experiments.

Following the convincing evidence that ${Ri}^{-3/2}$ should be expected in shear-free experiments, Long published two companion papers modifying his theory (Long 1978*a*,*b*). The former pertained to the velocity and length scale in a homogeneous fluid that predicted that

(1.8) $$\begin{array}{rl} u & ∼z^{-1}, \end{array}$$

(1.9) $$\begin{array}{rl} l & ∼z, \end{array}$$

consistent with observations. His modified theory for the stratified problem, discussed in Long (1978*a*), resulted in an entrainment rate given by

(1.10) $$\begin{array}{r} \frac{u_{e}}{u}∼{Ri}^{-\frac{7}{4}}, \end{array}$$

which is reasonably close to the three-halves power law considering the experimental scatter. The modified theory accounted for the inherent anisotropy of turbulence near the density interface and postulated that the vertical root-mean-square (RMS) velocity w , not u (the horizontal RMS velocity), should set the entrainment rate and w<u near the interface due to pressure-strain redistribution. Long (1978*a*) invoked rapid distortion theory (RDT) arguments of Hunt & Graham (1978) for turbulence impinging on a solid surface. The prediction (1.10) was corroborated in the experiments of Fernando & Long (1983, 1985).

Using state-of-the-art observational methods with a laser-light sheet and fluorescent dye in the non-turbulent layer, Hannoun, Fernando & List (1988) and Hannoun & List (1988) observed that at high Ri the primary entrainment mechanism is internal wave breaking. They developed an entrainment model based on this observation, incorporating the linear wave theoretical predictions of Phillips (1977), which is in agreement with the minus three-halves power law (Nokes 1988; Fernando 1991).

Nokes (1988), in an attempt to settle the debate on the entrainment dependence on Ri , ran a series of OGT experiments that led him to conclude that such a flow is extremely complex and that obtaining definitive answers is more challenging than originally thought. He found an entrainment exponent between −1.31 and −1.04 , significantly less than −1.5 and −1.75 supported by previous investigations. Nokes also concluded that a universal power law for the decay of the turbulence velocity scale was unlikely.

Fernando & Hunt (1997) developed what is probably the most sophisticated theory of turbulent entrainment based on the linearised governing equations. Their theory suggests that $u_{e}/u∼{Ri}^{-5/3}$ , reasonably close to ${Ri}^{-3/2}$ . Mcgrath, Fernando & Hunt (1997) ran a series of experiments at very large Richardson numbers and presented data that supported many of the theoretical predictions of Fernando & Hunt (1997).

Nokes (1988) discovered that beyond a certain stroke length, the flow became notably inhomogeneous with large-scale coherent jets meandering and contaminating the flow. Mcdougall (1979) also noted a surprising amount of horizontal heterogeneity of turbulence quantities that raised concerns about the reliability of predictions and empirical relationships derived from the assumption of planar homogeneity. McKenna & McGillis (2004) did a detailed study of flow repeatability and mean flow contamination in OGT experiments and found that turbulent fluctuations could vary by as much as 15 % between runs of the same geometry. They concluded that mean flow contamination and initial-condition sensitivity were inherent in such experiments and should be taken into account when doing such studies.

In light of these experimental challenges, numerical simulations become an attractive alternative to studying shear-free turbulence diffusion and its mixing characteristics at a density interface. It is therefore somewhat surprising that only two studies (known to us) investigate shear-free turbulent diffusion via numerical simulations. Briggs and coworkers ran a series of direct numerical simulations (DNS) and published the results in a set of companion papers, Briggs *et al.* (1996) and Briggs *et al.* (1998). Due to the relatively limited computational power available at the time, their simulations were necessarily at low Reynolds numbers. Their simulations confirmed the general consensus that entrainment at low *Ri* is due to large eddy engulfment of dense fluid and transitions to a ‘scouring’ regime for sufficiently high *Ri* where turbulent eddies wisp small amounts of dense fluid into the turbulent layer and entrainment proceeds at a very slow rate. They report a ${Ri}^{-3/2}$ entrainment rate in their simulations.

In the spirit of Briggs *et al.* (1996, 1998), we have run a series of high-resolution large eddy simulations (LES) of a flow that mimics the key attributes of the experimental set-up, namely, negligible mean shear and statistically one-dimensional distributions (in space). We are interested in investigating some of the classical questions surrounding this problem as well as some new ones. In particular, we want to answer the following questions.

- How do u and l scale with distance from the source region in the homogeneous fluid and can we determine an optimal definition for the virtual origin? See § 4.1.2 and Appendix C, respectively.
- How is the self-similarity of kinetic energy k and its dissipation rate ϵ , seen in the homogeneous fluid, modified by the presence of a stable density interface? See § 4.2.4.
- Can we determine scaling laws applicable in the stratified region? See § 4.2.4.
- How does the entrainment rate and density interface thickness scale with increasing stratification? See §§ 4.2.6 and 4.2.7, respectively.

The paper is organised as follows. Section 2 presents the governing equations, their non-dimensionalisation and statistical equations used in the analysis. Section 3 provides details on the numerical solution of the equations, computational domain, forcing protocol and subgrid stress (SGS) model used. Results are presented in § 4 where we seek to provide answers to the above questions. The results are synthesised and discussed in § 5 with a focus on turbulence modelling implications.

## 2. Governing equations

The numerical code used in this paper solves the filtered incompressible Navier–Stokes equations under the Boussinesq approximation (Spiegel & Veronis 1960). In dimensional form, these are given as

(2.1a) $$\begin{array}{rl} & \frac{\partial u_{\;j}^{*}}{\partial x_{\;j}^{*}}=0, \end{array}$$

(2.1b) $$\begin{array}{rl} & \frac{Du_{i}^{*}}{Dt^{*}}=-\frac{1}{\rho_{r}^{*}}\frac{\partial p_{nh}^{*}}{\partial x_{i}^{*}}-\frac{\rho_{nh}^{*}}{\rho_{r}^{*}}g^{*}\delta_{i3}+\nu^{*}\frac{\partial^{2}u_{i}^{*}}{\partial x_{\;j}^{*}\partial x_{\;j}^{*}}-\frac{\partial \tau_{\text{ij}}^{*}}{\partial x_{\;j}^{*}}+S_{i}^{*}, \end{array}$$

(2.1c) $$\begin{array}{rl} & \frac{DT^{*}}{Dt^{*}}=\kappa^{*}\frac{\partial^{2}T^{*}}{\partial x_{\;j}^{*}\partial x_{\;j}^{*}}-\frac{\partial q_{j}^{*}}{\partial x_{\;j}^{*}}+S_{T}^{*}, \end{array}$$

where $u_{i}^{*}$ is the i th component of the velocity vector and i∈{1,2,3} . Alternatively, $u^{*}$ , $v^{*}$ and $w^{*}$ can be used in place of $u_{1}^{*}$ , $u_{2}^{*}$ and $u_{3}^{*}$ . These components are defined in an orthogonal Cartesian coordinate system with $x_{1}^{*}$ , $x_{2}^{*}$ , $x_{3}^{*}$ denoting the three independent spatial dimensions, equivalently denoted as $x^{*}$ , $y^{*}$ and $z^{*}$ . Here $\rho^{*}$ is the fluid density, $p^{*}$ is pressure, $\kappa^{*}$ is the thermal diffusivity, $\nu^{*}\equiv \mu^{*}/\rho_{r}^{*}$ is the kinematic viscosity with $\mu^{*}$ the molecular dynamic viscosity and $g^{*}=-g^{*}\delta_{i3}$ is the gravitational acceleration vector pointing in the negative z direction; $\tau_{\text{ij}}^{*}$ and $q_{j}^{*}$ are the SGS tensor and heat flux vector due to filtering the governing equations. Closure of these terms and grid convergence of the solution will be discussed in more detail below. The momentum and heat source terms $S_{i}^{*}$ and $S_{T}^{*}$ are also discussed in detail later in this section. Quantities with an asterisk are dimensional. Here $Df^{*}/Dt^{*}=(\partial_{t^{*}}+u_{\;j}^{*}\partial_{j})f$ is the material derivative. The Einstein summation convention for repeated indices is used throughout unless otherwise specified.

Thermodynamic state variables are decomposed as

(2.2) $$\begin{array}{rl} f^{*}(x,y,z,t) & =f_{r}^{*}+f_{h}^{*}(z)+f_{nh}^{*}(x,y,z,t), \end{array}$$

where $f^{*}\in \{\rho^{*},p^{*},T^{*}\}$ generically represents any of the state variables. Here $f_{r}^{*}$ is a constant reference value, $f_{h}^{*}(z)$ is the hydrostatic base state and $f_{nh}^{*}(x,y,z,t)$ is the non-hydrostatic component resulting from bulk fluid motion. Pressure and density in the absence of fluid motion satisfy the hydrostatic balance equation and, hence, only their non-hydrostatic components appear in the momentum equation.

Under the conditions that permit the Boussinesq approximation, density and temperature are related via the linearised equation of state (Spiegel & Veronis 1960)

(2.3) $$\begin{array}{rl} \frac{\rho^{*}-\rho_{r}^{*}}{\rho_{r}^{*}} & =-\alpha_{r}^{*}(T^{*}-T_{r}^{*}), \end{array}$$

where $\alpha_{r}^{*}\equiv [-1/\rho^{*}(\partial \rho^{*}/\partial T^{*}){]}_{r}$ is the coefficient of thermal expansion at the reference state.

### 2.1. Non-dimensional equations

The velocity, length and temperature scales chosen to non-dimensionalise (2.1) are $U^{*}$ , $L^{*}$ and $\Delta T^{*}$ . Here $U^{*}$ and $L^{*}$ are characteristic scales of the forcing layer turbulence; $\Delta T^{*}$ is the initial temperature jump across the density interface. The resulting non-dimensional equations are

(2.4a) $$\begin{array}{rl} & \frac{\partial u_{\;j}}{\partial x_{\;j}}=0, \end{array}$$

(2.4b) $$\begin{array}{rl} & \frac{Du_{i}}{Dt}=-\frac{\partial p_{nh}}{\partial x_{i}}+\frac{T_{nh}}{{Fr}^{2}}\delta_{i3}+\frac{1}{\text{Re}}\frac{\partial^{2}u_{i}}{\partial x_{\;j}\partial x_{\;j}}-\frac{\partial \tau_{\text{ij}}}{\partial x_{\;j}}+S_{i}, \end{array}$$

(2.4c) $$\begin{array}{rl} & \frac{DT}{Dt}=\frac{1}{Pr\text{Re}}\frac{\partial^{2}T}{\partial x_{\;j}\partial x_{\;j}}-\frac{\partial q_{j}}{\partial x_{\;j}}+S_{T}, \end{array}$$

where $\text{Re}=U^{*}L^{*}/\nu^{*}$ is the Reynolds number, $\text{Pr}=\nu^{*}/\kappa^{*}$ the Prandtl number, $\text{Fr}=U^{*}/\sqrt{\Delta b^{*}L^{*}}$ the Froude number and $\Delta b^{*}=\alpha_{r}^{*}g^{*}\Delta T^{*}$ is the buoyancy jump across the interface. Note that the heat equation (2.4*c*) is for the total temperature T , not just the non-hydrostatic component. In other words, we do not force a particular hydrostatic profile, but rather it is an outcome of the flow evolution.

### 2.2. Statistical equations

Reference to a number of statistical quantities and equations will be made in subsequent sections. We present and define all such quantities here. A Reynolds decomposition is used to separate the flow variables into a mean and fluctuating component: $f=\langle f\rangle +f^{\prime }$ . Unless otherwise specified, angle brackets denote a horizontal and time average. Given the forcing protocol described below, $\langle u_{i}\rangle ≃0$ and $u_{i}^{\prime }≃u_{i}$ .

Multiplying (2.4*b*) by $u_{i}^{\prime }$ and averaging, one can derive the equation for TKE, k :

(2.5a) $$\begin{array}{rl} \frac{\partial k}{\partial t} & =\frac{\partial F}{\partial z}-B-\epsilon +F_{k}, \end{array}$$

(2.5b) $$\begin{array}{rl} k & =\langle u_{i}^{\prime }u_{i}^{\prime }\rangle /2, \end{array}$$

(2.5c) $$\begin{array}{rl} F & =-\langle w^{\prime }u_{i}^{\prime }u_{i}^{\prime }\rangle /2-\langle w^{\prime }p^{\prime }\rangle +\frac{2}{\text{Re}}\langle u_{i}^{\prime }s_{\text{ij}}^{\prime }\rangle -\langle u_{i}^{\prime }\tau_{\text{ij}}\rangle , \end{array}$$

(2.5d) $$\begin{array}{rl} B & =-\frac{1}{{Fr}^{2}}\langle w^{\prime }T^{\prime }\rangle , \end{array}$$

(2.5e) $$\begin{array}{rl} \epsilon & =\frac{2}{\text{Re}}\langle s_{\text{ij}}^{\prime }s_{\text{ij}}^{\prime }\rangle -\langle \tau_{\text{ij}}s_{\text{ij}}^{\prime }\rangle , \end{array}$$

(2.5f) $$\begin{array}{rl} F_{k} & =\langle u_{i}^{\prime }S_{i}^{\prime }\rangle , \end{array}$$

where F is the kinetic energy flux, B is the buoyancy flux, ϵ is the TKE dissipation rate (or simply the ‘dissipation rate’ or the ‘dissipation’), $F_{k}$ is the work due to forcing and $s_{\text{ij}}=(1/2)((\partial u_{i}/\partial x_{\;j})+(\partial u_{\;j}/\partial x_{i}))$ is the strain rate tensor.

The symbol ‘ l ’ in the introduction referred to the integral length scale for historical purposes (that is, the symbol preferred by most authors reporting on OGT experiments). Moving forward, we reserve the symbol l for the turbulence length scale:

(2.6) $$\begin{array}{r} l=\frac{k^{\frac{3}{2}}}{\epsilon }. \end{array}$$

Integrating (2.5*a*) over z gives an equation for the total kinetic energy (recall that the mean kinetic energy is equal to zero):

(2.7a) $$\begin{array}{rl} \frac{\partial K}{\partial t} & =\Delta F-B-\epsilon_{K}+F_{K}, \end{array}$$

(2.7b) $$\begin{array}{rl} K & =\int_{z_{b}}^{z}kdz, \end{array}$$

(2.7c) $$\begin{array}{rl} \Delta F & =F(z)-F(z_{b}), \end{array}$$

(2.7d) $$\begin{array}{rl} B & =\int_{z_{b}}^{z}B(s)ds, \end{array}$$

(2.7e) $$\begin{array}{rl} \epsilon_{K} & =\int_{z_{b}}^{z}\epsilon (s)ds, \end{array}$$

(2.7f) $$\begin{array}{rl} F_{K} & =\int_{z_{b}}^{z}F_{k}(s)ds. \end{array}$$

Multiplying (2.4*c*) by $1/{Fr}^{2}$ and averaging yields an equation for the mean buoyancy:

(2.8a) $$\begin{array}{rl} \frac{\partial \langle b\rangle }{\partial t} & =\frac{\partial B}{\partial z}+\frac{\partial }{\partial z}[\frac{1}{\text{Pr}\text{Re}}N^{2}-\frac{\left\langle q_{3}\right\rangle }{{\text{Fr}}^{2}}]+\frac{\left\langle S_{T}\right\rangle }{{\text{Fr}}^{2}}, \end{array}$$

(2.8b) $$\begin{array}{rl} N^{2} & =\frac{\partial \langle b\rangle }{\partial z}. \end{array}$$

Here $b\equiv T/{Fr}^{2}$ is the non-dimensional buoyancy and N the buoyancy frequency. Multiplying (2.8) by −z and integrating in z gives an equation for the total potential energy:

(2.9a) $$\begin{array}{rl} \frac{\partial P}{\partial t} & =B+\Delta F_{P}-\epsilon_{P}-S_{P}, \end{array}$$

(2.9b) $$\begin{array}{rl} P & =\int_{z_{b}}^{z}-(s-z_{b})\langle b\rangle (s)ds, \end{array}$$

(2.9c) $$\begin{array}{rl} \Delta F_{P} & =[-sB(s)-\frac{1}{Pr\text{Re}}\frac{\partial (s\langle b\rangle )}{\partial s}+s\frac{\left\langle q_{3}\rangle (s\right)}{{Fr}^{2}}{]}_{z_{b}}^{z}, \end{array}$$

(2.9d) $$\begin{array}{rl} \epsilon_{P} & =-\frac{2}{Pr\text{Re}}(\langle b\rangle (z)-\langle b\rangle (z_{b}))+\int_{z_{b}}^{z}\frac{\left\langle q_{3}\rangle (s\right)}{{Fr}^{2}}ds, \end{array}$$

(2.9e) $$\begin{array}{rl} S_{P} & =\int_{z_{b}}^{z}(s-z_{b})\frac{\left\langle S_{T}\rangle (s\right)}{{Fr}^{2}}ds. \end{array}$$

Potential energy in (2.9*b*) is defined relative to a reference height taken to be $z_{b}$ , the bottom of our computational domain.

**Figure 1.**
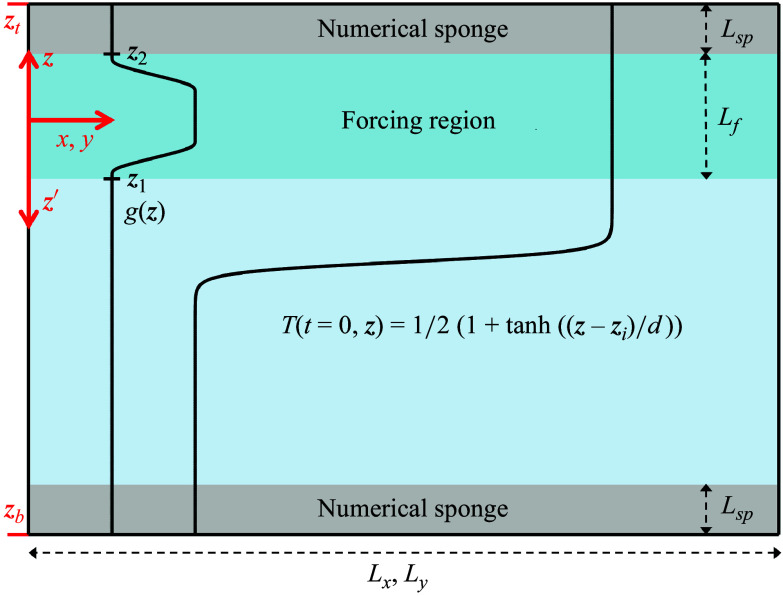
Figure 1 long description. Schematic of computational domain. Note that $z^{\prime }$ is defined positive downward. Everything in the schematic is to scale for the stratified simulations, i.e. $L_{x}/(z_{t}-z_{b})=1.5$ as opposed to 1 for the unstratified cases.

## 3. Problem set-up

Equations (2.4) are integrated in a high-order code developed in our group that uses Fourier collocation in the homogeneous plane ( x and y directions) and sixth-order compact finite differencing in the z direction (Ghate 2018). The nonlinear terms are dealiased (in the horizontal plane) by zeroing one-third of Fourier components at the high wavenumber end. The compact schemes are formulated using a staggered configuration: vertical velocity is stored at the faces of computational cells while pressure, scalar concentration and horizontal velocities are stored at cell centres.

### 3.1. Computational domain

A schematic of the domain is shown in figure 1. Dimensions of the domain for each case are given in table 2. The smallest domain is used for the unstratified runs, which is a cube with sides of (non-dimensional) length 6 that is large enough to capture about three large-scale eddies near the bottom of the domain as measured by $L_{11}^{\prime }$ (as discussed in the introduction, the length scale grows with distance from the source region so it is the bottom of the domain where $L_{11}^{\prime }$ is maximum):

(3.1) $$\begin{array}{rl} f(r_{1}=L_{11}^{\prime },z) & =0.1, \end{array}$$

(3.2) $$\begin{array}{rl} f(r_{1},z) & =\frac{\left\langle u^{\prime }(x,y,z,t)u^{\prime }(x+r_{1},y,z,t)\right\rangle }{\left\langle u^{\prime 2}(x,y,z,t)\right\rangle }. \end{array}$$

**Table 2.** Domain size parameters. See figure 1 for definitions. There are two levels of mesh resolutions considered: medium (M) and fine (F). These represent mesh spacings of 3/128 and 3/256 , respectively. Statistics reported in this paper are computed from F-mesh results. However, all F-mesh simulations are initialised from M-mesh runs to efficiently bypass the early transient (propagation of the turbulent front from the forcing region to the density interface).

Run ID	$L_{x}$ , $L_{y}$	$z_{b}$	$z_{t}$	$L_{sp}$	$L_{\;f}$	$N_{x}$ , $N_{y}$	$N_{z}$	Resolution level
SM71, SM72, SM73, SM74	6	−4	2	0.6	1.5	512	512	F
SM123, SM124, SM125, SM126	9	−5.025	1.35	0.6	1.5	384	272	M
						768	544	F
SM130	9	−5.025	1.35	0.6	1.5	384	272	M

In other words, $L_{11}^{\prime }$ is the value of $r_{1}$ where f=0.1 . We chose this particular definition of the longitudinal length scale over the traditional integral definition because the integral of $f(r_{1})$ (up to a cutoff location of f=0.1 ) is significantly smaller than $L_{11}^{\prime }$ and it was observed (by overlaying both definitions on instantaneous velocity contours) that $L_{11}^{\prime }$ is more representative of the large eddies. For the stratified runs, we used domains that were 50 % larger in the horizontal dimensions to allow for amplification of the horizontal length scales near the density interface without artificially confining the flattened eddies; this larger horizontal domain is depicted in figure 1.

Fluctuations are smoothly damped out in sponge regions at the top and bottom of the domain by adding a penalty term to the momentum equation $S_{i}^{\left(sp\right)}=(r(z)/\tau_{sp})(0-u_{i})$ , forcing velocity to zero. Here r(z) is a cosine ramp going from 0 to 1 across the sponge region and $\tau_{sp}$ is a user-specified time scale characterising the rate of damping. All cases used $L_{sp}=0.6$ for the sponge layer thickness, which was sufficient to damp out all fluctuations before reaching the domain boundary. (We want to emphasise that internal gravity waves are confined to the interfacial region, i.e. they do not propagate vertically. Therefore, a thin sponge region is suitable for our purposes.)

The forcing layer thickness was set to $L_{\;f}=1.5$ for all cases. To compare $L_{\;f}$ to physical scales, we define the turbulence length scale in the forcing region $l_{f}=k_{\;f}^{3/2}/\epsilon_{\;f}$ , with $k_{\;f}=\langle k\rangle_{\;f}$ the forcing layer TKE and $\epsilon_{\;f}=\langle \epsilon \rangle_{\;f}$ the dissipation of $k_{\;f}$ , where angle brackets $\langle \cdot \rangle_{\;f}$ denote a vertical average over the forcing region. The forcing layer thickness is 4.4 times the turbulence length scale in the forcing layer (i.e. $L_{\;f}/l_{f}=4.4$ ); running a 1.5 times larger forcing region showed no discernible change in statistical quantities. A discussion of sensitivity to the domain size, forcing layer thickness and forcing and sponge layer proximity is provided in Appendix A, with more details available in Hass (2025).

### 3.2. Turbulence forcing protocol

Turbulence is generated in a localised region of space by adding a source term ( $S_{i}=A_{\text{ij}}(t)f_{\;j}$ ) to the momentum equations. There are two features of the source term: a time-dependent controller $A_{\text{ij}}$ and a momentum forcing term $f_{\;j}$ . Inspired by the simulations of Briggs *et al.* (1996, 1998) the forcing term is specified as

(3.3) $$\begin{array}{r} {\hat{f}}_{i}(\alpha ,\beta ,z)=[g(z)h(\kappa_{h}){\hat{u}}_{i}(\alpha ,\beta ,z){]}^{⊥}, \end{array}$$

where a ‘hat’ accent ( $\hat{\cdot }$ ) denotes the horizontal Fourier transform of a quantity, α and β are the wave vector components in the x and y directions, respectively, and $\kappa_{h}=\sqrt{\alpha^{2}+\beta^{2}}$ is the horizontal wavenumber. The ⊥ superscript emphasises that once the forcing term is constructed it is projected onto a divergence-free basis, explicitly removing its pressure contribution. The masking function g(z) provides spatial locality and defines the thickness of the forcing region $L_{\;f}$ :

(3.4a) $$\begin{array}{rl} g(z) & =S(\frac{z-z_{1}}{d_{1}})-S(\frac{z-z_{2}}{d_{2}}+1), \end{array}$$

(3.4b) $$\begin{array}{rl} S(\zeta ) & =\left\{ \begin{array}{ll} 0 & \text{for }\zeta ⩽0,\\ 1 & \text{for }\zeta ⩾1,\\ {}[1+\exp\left(1/(\zeta -1)+1/\zeta \right){]}^{-1} & \text{otherwise}, \end{array}\right. \end{array}$$

(3.4c) $$\begin{array}{rl} L_{\;f} & \equiv z_{2}-z_{1}. \end{array}$$

**Figure 2.**
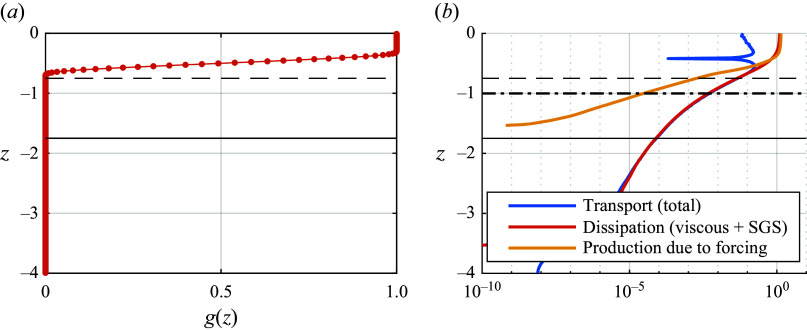
Figure 2 long description. Forcing layer localisation. (*a*) Forcing layer mask function, g(z) . (*b*) Absolute value of the dominant terms in the TKE budget, showing the rapid decay of the source term outside the forcing layer for run SM74. Below z=−1 (black dash–dot line) TKE production due to forcing is virtually negligible. To show the proximity of the density interface (stratified runs only) and the forcing region, a thin black line denotes $z_{i}$ in (3.9); dashed black lines in (*a*) and (*b*) mark the edge of the forcing region, $z_{2}$ , in (3.4*a*).

The choice $L_{\;f}=1.5$ (i.e. $z_{1}=-0.75$ , $z_{2}=0.75$ ) and $d_{1}=d_{2}=0.5$ results in the g(z) overlaid in figure 1 and shown in figure 2(*a*). Figure 2(*b*) demonstrates that outside the forcing region, turbulence production due to the forcing term is negligible compared with the other terms in the TKE budget (2.5*a*).

The spectral mask $h(\kappa_{h})$ in (3.3) determines the wavenumber band that is forced and is given by

(3.5) $$\begin{array}{r} h(\kappa )=\left\{ \begin{array}{ll} 1 & \text{for }\;\kappa_{\text{min}}⩽\kappa ⩽\kappa_{\text{max}},\\ 0 & \text{otherwise}. \end{array}\right. \end{array}$$

Following Briggs *et al.* (1998) we set $\kappa_{\text{max}}=45$ , ensuring the forcing is applied only to the large scales and a physical cascading process transfers energy to the small scales. The non-dimensional length scale characterising the forcing layer turbulence is set by $\kappa_{\text{min}}$ , which we systematically varied (by a factor of two) to determine the role of this parameter.

The kinetic energy (or velocity scale) in the forcing region is set by the controller $A_{\text{ij}}$ , which extends the ideas of Bassenne *et al.* (2016) to a localised region of space. Bassenne *et al.* (2016), interested in studying forced homogeneous isotropic turbulence, proposed a controller $A(t)=(\epsilon (t)-(G/\tau )(k(t)-k_{\infty })/(2k(t))$ that efficiently pushes the simulation to a target TKE, $k_{\infty }$ . The form of A ensures the kinetic energy converges exponentially fast to the target: $dk/dt=-\epsilon +2Ak=-G(k-k_{\infty })/\tau$ . In our context, we have the complication of inhomogeneous transport terms in the TKE equation and generalise the approach to an arbitrary forcing vector $f_{i}$ . Specifically, $A_{\text{ij}}$ is defined as

(3.6a) $$\begin{array}{rl} A_{\text{ij}} & =\left[\begin{array}{ccc} A_{x} & 0 & 0\\ 0 & A_{y} & 0\\ 0 & 0 & A_{z} \end{array}\right], \end{array}$$

(3.6b) $$\begin{array}{rl} A_{\alpha }(t) & =\frac{{\tilde{\epsilon }}_{\alpha ,V}(t)-\Delta F_{\alpha }-\frac{G}{\tau }[k_{\alpha ,V}(t)-k_{\text{tgt}}/3]}{f_{\alpha ,V}(t)}. \end{array}$$

The terms in (3.6*b*) come from component-wise TKE equations analogous to (2.5) which are vertically averaged over the forcing region. These are given by,

(3.7a) $$\begin{array}{rl} \frac{\partial k_{\alpha ,V}}{\partial t} & =\Delta F_{\alpha }-{\tilde{\epsilon }}_{\alpha ,V}+A_{\alpha j}(t)f_{\;j,V}, \end{array}$$

(3.7b) $$\begin{array}{rl} \Delta F_{\alpha } & =F_{\alpha }(z_{2})-F_{\alpha }(z_{1}), \end{array}$$

(3.7c) $$\begin{array}{rl} F_{\alpha } & =-\langle w^{\prime }u_{\alpha }^{\prime 2}\rangle /2-\langle w^{\prime }p^{\prime }\rangle \delta_{\alpha 3}+\frac{1}{\text{Re}}\langle u_{\alpha }^{\prime }\frac{\partial u_{\alpha }^{\prime }}{\partial z}\rangle -\langle u_{\alpha }^{\prime }\tau_{\alpha 3}^{\text{SGS}}\rangle , \end{array}$$

(3.7d) $$\begin{array}{rl} {\tilde{\epsilon }}_{\alpha ,V}(t) & =\frac{1}{V}\int_{V}(\frac{1}{Re}+\nu_{\text{SGS}})\frac{\partial u_{\alpha }^{\prime }}{\partial x_{\;j}}\frac{\partial u_{\alpha }^{\prime }}{\partial x_{\;j}}dV, \end{array}$$

(3.7e) $$\begin{array}{rl} k_{\alpha ,V}(t) & =\frac{1}{V}\int_{V}u_{\alpha }^{\prime }u_{\alpha }^{\prime }/2dV, \end{array}$$

(3.7f) $$\begin{array}{rl} f_{\alpha ,V}(t) & =\frac{1}{V}\int_{V}u_{\alpha }^{\prime }f_{\alpha }^{\prime }dV. \end{array}$$

Here $k_{\alpha }$ is the α component of the TKE, $F_{\alpha }$ is the flux of $k_{\alpha }$ , ${\tilde{\epsilon }}_{\alpha }$ is the pseudo dissipation of $k_{\alpha }$ , $f_{\alpha }$ is the production of $k_{\alpha }$ due to forcing and $\nu_{\text{SGS}}$ is used to close the subgrid stress tensor and is discussed in § 3.4. The subscript ‘ V ’ denotes a volume average over the forcing region.

Equation (3.6*b*) can be derived by insisting that $\partial_{t}k_{\alpha ,V}=0$ . (In practice, $\Delta F_{\alpha }$ (3.6*b*) is omitted in our code as we found that it was much smaller than the other terms and the forcing layer turbulence quickly reached a stationary state when neglecting it.) There is no buoyancy term in (3.7*a*) since the forcing region is of uniform density. We used a constant value of G/τ=10 for all cases and found that this quickly converged to the target kinetic energy level.

For a fixed choice of g(z) and G/τ , the remaining input parameters available to the user are $\kappa_{\text{min}}$ in (3.5) and $k_{\text{tgt}}$ in (3.6*b*), effectively setting the length and velocity scale in the forcing region. Values used in our simulations are reported in table 3.

**Table 3.** Input and output parameters of the simulations. Here Re and Fr depend on the reference length and velocity scales, $L^{*}$ and $U^{*}$ . More meaningful output parameters are reported as well where ${\text{Re}}_{t}=\text{Re}k^{2}/\epsilon$ , ${\text{Fr}}_{t}=\epsilon /Nk$ , ${\text{Re}}_{b}=\text{Re}\epsilon /N^{2}$ and $N^{2}=\partial_{z}\langle T\rangle /{Fr}^{2}$ . We define $z_{I}$ as the location where ⟨T⟩=0.5 or $z_{I}=-1.75$ for the unstratified runs (i.e. $z_{i}$ in (3.9)). The output parameters are computed from time and planar averages. Run SM130 is unstratified (i.e. Fr=∞ ), but was integrated along with the (passive) scalar evolution equation and so scalar evolution can be compared with the stratified runs. Scalar fields were not considered in the other unstratified simulations. The last column documents the amount of time averaging used to compute statistics in terms of $\tau_{0.7}$ , the eddy turnover time where ⟨T⟩=0.7 (stratified runs) or where $z^{\prime }=1.75$ (unstratified simulations).

	Input parameters					Output parameters				Averaging
Run ID	Re	Fr	Pr	$k_{\text{tgt}}$	$\kappa_{\text{min}}$	Maximum	${\text{Fr}}_{t}(z_{I})$	${\text{Re}}_{t}(z_{I})$	${\text{Re}}_{b}(z_{I})$	window
						${\text{Re}}_{t}(z)$				$t_{avg}/\tau_{0.7}$
SM71	100 000	—	—	10	14	10 199	—	419	—	2.90
SM72	100 000	—	—	20	14	14 469	—	676	—	2.71
SM73	100 000	—	—	10	7	17 570	—	970	—	5.23
SM74	100 000	—	—	20	7	24 694	—	1,659	—	4.90
SM130	100 000	∞	7.2	20	7	22 913	∞	1,717	—	1.82
SM123	100,000	14	7.2	20	7	25 319	0.568	—	425	6.83
SM124	100 000	7	7.2	20	7	25 462	0.227	—	99.5	5.80
SM125	100 000	3.5	7.2	20	7	25 359	0.082	—	16.3	5.79
SM126	100 000	1.25	7.2	20	7	25 397	0.009	—	1.01	2.49

A heat source is added to the temperature equation for two reasons: first, the non-dimensional temperature remains bounded between zero and one between the forcing layer edge and the bottom of the domain, and second, by ensuring a uniform temperature in the forcing layer there is no heat flux through it so the momentum forcing protocol does not interact directly with the scalar being mixed. The source is defined as

(3.8) $$\begin{array}{r} S_{T}=\frac{g(z)}{\tau_{T}}(1-T), \end{array}$$

where g(z) is that defined in (3.4*a*), $\tau_{T}$ is the relaxation time scale and is taken to be $10\Delta_{t}$ in all simulations in this work where $\Delta_{t}$ is the time-step size. We explored other values of $\tau_{T}$ and chose $10\Delta_{t}$ , which gives a balance between being well resolved in time and results in a very thin zone (in z ) over which the heat source is applied giving a well-defined edge to the forcing layer.

### 3.3. Initial density interface location and thickness

The temperature initial condition is

(3.9) $$\begin{array}{rl} T(t=0) & =\frac{1}{2}[1+\tanh(\frac{z-z_{i}}{d})], \end{array}$$

where $z_{i}$ is the location of the stratification interface and d is half the initial interface thickness. In every simulation $z_{i}=-1.75$ and d=0.125 . The density interface location was chosen to maximise the available grid resolution (i.e. reduce the amount of SGS dissipation in the interfacial region) while also minimising the length of integration time required to achieve a reasonable statistical estimate because the eddy turnover time τ=k/ϵ grows as the square of the distance from the forcing region. This results in a significant time-scale separation from the large eddies near the interface and the Courant–Friedrichs–Lewy (CFL) condition set by the Nyquist scale. Here $z_{i}$ is depicted as solid black lines in figure 2. (The particular forcing mask visualised in figures 2(*a*) and 2(*b*) is for the domain used in the unstratified simulations, i.e. z∈[−4,2] . The stratified runs used a slightly different domain ( z∈[−5.025,1.35] ), but the forcing layer thickness and proximity to the initial density interface is the same in every case.) This location corresponds to a distance of $2.04l_{i}$ from the forcing layer edge, where $l_{i}$ is the large eddy length scale (in the homogeneous fluid simulations) at $z_{i}$ , i.e. $l_{i}=l(z_{i})$ ; in terms of $l_{\;f}$ the distance is $2.91l_{\;f}$ . The value of d in (3.9) was chosen to approximate an initial two-layer system while still adequately resolving the interface. This compromise is discussed in more detail in § 4.2.3.

### 3.4. The SGS closure and grid convergence of the solutions

Formally, $\tau_{\text{ij}}$ in (2.4*b*) is the deviatoric component of the tensor ${\tilde{\tau }}_{\text{ij}}\equiv {\bar{u_{i}u}}_{j}-{\bar{u}}_{i}{\bar{u}}_{j}$ , where the overline denotes a filtering operation implied by the LES methodology (Pope 2001). The trace of ${\tilde{\tau }}_{\text{ij}}$ is absorbed into the pressure and not modelled explicitly. Similarly, the subfilter (or subgrid in the LES context) heat flux $q_{j}\equiv \bar{u_{\;j}T}-{\bar{u}}_{j}\bar{T}$ . Note that (2.4*b*) are for the filtered velocity ${\bar{u}}_{i}$ and temperature $\bar{T}$ fields where the overline has been omitted and will not be included moving forward.

The SGS tensor $\tau_{\text{ij}}$ is closed via the constant-coefficient ‘Sigma’ model of Nicoud *et al.* (2011). We note that the particular closure model used should not significantly influence results since the mesh resolution achieves 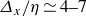 in the interfacial region (see Appendix A), which is the region of interest; $\eta =1/({\text{Re}}^{3}\epsilon {)}^{1/4}$ being the non-dimensional Kolmogorov length scale. (Recall that quantities without an asterisk are dimensionless as discussed in § 2.1.) Furthermore, the Sigma model has a long track record of reliable results for homogeneous isotropic (unstratified) turbulence, which characterises the flow in the forcing region and well above the density interface where the Nyquist length scale is much larger than η .

A necessary and sufficient condition for obtaining accurate mixing statistics in LES of stratified flows is that the mesh spacing $\Delta_{x}⩽L_{O}$ (Khani 2018), where $L_{O}=(\epsilon /N^{3}{)}^{1/3}$ is the Ozmidov length scale and $N=\sqrt{\partial_{z}\langle T\rangle }/\text{Fr}$ the non-dimensional buoyancy frequency. This precaution ensures that all scales modelled by the SGS model are isotropic and unaffected by internal gravity waves. This requirement is met for all simulations in the present paper with the exception of the most highly stratified case where $\Delta_{x}/L_{O}⩽4$ and the region where $\Delta_{x}/L_{O}>1$ is confined to a thin zone near the interface (see figure 30 in Appendix A).

A detailed mesh sensitivity study was conducted and reported in Appendix A showing that the statistical quantities of interest are grid converged for the resolutions used in this paper. Finally, we note that some previous LES studies of stratified flows do show sensitivity to the particular SGS closure (see, e.g. Bohnert 1993 and references therein). However, in these studies the gap between the Nyquist and molecular scales is much larger than in the present work (and the Ozmidov scale is almost certainly unresolved). In such under-resolved simulations it is completely expected that the results would show strong sensitivity to the choice of closure model.

We use $\nu_{\text{SGS}}/\kappa_{\text{SGS}}=Pr_{t}=0.9$ to relate the scalar and momentum subgrid fluxes. Use of a constant value for ${\text{Pr}}_{t}$ is common in the literature with values ranging from 0.3–1.0 (see, e.g. Siegel & Domaradzki 1994; Khani & Waite 2014; Matheou & Chung 2014).

It is well known that LES of the scalar-transport equation results in unphysical oscillations above and below the physical bounds set by initial and boundary conditions (Sharan, Matheou & Dimotakis 2018). We have chosen to address this by modifying our SGS model to apply additional diffusion locally in regions where the scalar is out of bounds following the ideas of Cook (2007). The specific form of the modified SGS scalar flux is given by

(3.10a) $$\begin{array}{rl} q_{j}^{\text{SGS}} & =-\tilde{\kappa }\frac{\partial T}{\partial x_{\;j}}, \end{array}$$

(3.10b) $$\begin{array}{rl} \tilde{\kappa } & =[1-H(\kappa_{\text{SB}}-\kappa_{\text{th}})]\kappa_{\text{SGS}}+H(\kappa_{\text{SB}}-\kappa_{\text{th}})\kappa_{\text{SB}}, \end{array}$$

(3.10c) $$\begin{array}{rl} \kappa_{\text{SGS}} & =\frac{\nu_{\text{SGS}}}{{\text{Pr}}_{t}}, \end{array}$$

(3.10d) $$\begin{array}{rl} \kappa_{\text{SB}} & =C_{y}\bar{(\frac{\delta^{2}}{\Delta t_{CFL}}\eta )}, \end{array}$$

(3.10e) $$\begin{array}{rl} \eta & =|T|-1+|1-T|, \end{array}$$

(3.10f) $$\begin{array}{rl} \delta & =\frac{\Delta_{i}|\partial_{i}T|}{\sqrt{\partial_{j}T\partial_{j}T}}, \end{array}$$

where $C_{y}=10$ , $\Delta_{i}$ is the mesh spacing, H is the Heaviside function, $\Delta t_{CFL}$ is the CFL condition given by $\min_{x}\{(|u|/\Delta_{x}+|v|/\Delta_{y}+|w|/\Delta_{z}{)}^{-1}\}$ and the overline $\bar{\left(\cdot \right)}$ denotes a discrete Gaussian filter. Further details of the procedure are presented in Appendix B. Recall that temperature is non-dimensionalised to be bounded between zero and one, so (3.10*e*) is non-zero only when T is outside of this range.

### 3.5. Parameter space

Having introduced the non-dimensional equations, forcing protocol and computational domain, we can now present the parameter space explored by our simulations. This is summarised in table 3.

The input parameters Re , Fr , $k_{\text{tgt}}$ and $\kappa_{\text{min}}$ result in a flow that can be characterised by three output non-dimensional parameters: a turbulent Reynolds number ${\text{Re}}_{t}$ , turbulent Froude number ${\text{Fr}}_{t}$ and the buoyancy Reynolds number ${\text{Re}}_{b}$ . In terms of dimensionless variables (see § 2.1) these are defined as

(3.11) $$\begin{array}{rl} {\text{Re}}_{t} & =\text{Re}u_{c}l_{c}, \end{array}$$

(3.12) $$\begin{array}{rl} {\text{Fr}}_{t} & =\frac{u_{c}}{Nl_{c}}, \end{array}$$

(3.13) $$\begin{array}{rl} {\text{Re}}_{b} & =\text{Re}\frac{\epsilon }{N^{2}}, \end{array}$$

where $u_{c}$ and $l_{c}$ are characteristic velocity and length scales of the turbulence. If $\epsilon ∼u_{c}^{3}/l_{c}$ then ${\text{Fr}}_{t}^{2}{\text{Re}}_{t}∼{\text{Re}}_{b}$ , demonstrating that only two are independent. There are good reasons to prefer using ${\text{Re}}_{t}$ and ${\text{Fr}}_{t}$ to fully characterise the turbulence (Ivey & Imberger 1991; Mater & Venayagamoorthy 2014) though ${\text{Re}}_{b}$ is still widely used in the stratified turbulence literature. Here ${\text{Re}}_{t}$ is the ratio of inertial-to-viscous forces; ${\text{Fr}}_{t}$ is a time-scale ratio between the buoyancy time scale $\tau_{b}=1/N$ and the turbulence time scale $\tau_{t}=l_{c}/u_{c}$ ; and $\sqrt{{\text{Re}}_{b}}$ is a ratio between the buoyancy and viscous time scales, $\tau_{b}$ and $\tau_{\eta }=\sqrt{1/\epsilon \text{Re}}$ , respectively. When ${\text{Fr}}_{t}<1$ , buoyancy sets the dynamic time scale rather than the large-scale turbulence. In this case, it is ${\text{Re}}_{b}$ that describes the dynamic range of inertial eddies rather than ${\text{Re}}_{t}$ and, hence, it is instructive to consider both ${\text{Re}}_{t}$ and ${\text{Re}}_{b}$ when interpreting their numerical value.

By defining $u_{c}=\sqrt{k}$ and $l_{c}=k^{3/2}/\epsilon$ , each non-dimensional parameter can be interpreted as a length-scale ratio (Caulfield 2021; Yi & Koseff 2022). For now, the above interpretation suffices to appreciate the values reported in table 3. The maximum ${\text{Re}}_{t}$ occurs in the forcing layer where there is no stratification. Here ${\text{Fr}}_{t}$ is reported at the interface midpoint $z_{I}$ where stratification affects are dominant. We also report ${\text{Re}}_{t}$ at the initial interface location for the unstratified runs to give a sense of the turbulence level in the interfacial region, absent buoyancy effects. Similarly, we report ${\text{Re}}_{b}$ at the interface location for the stratified runs where the time-scale separation between buoyancy and viscosity gives a sense of how vigorous turbulence is at that location. (Here ${\text{Re}}_{t}(z_{I})$ for the stratified cases is significantly affected by interfacial waves and so does not give a meaningful description of the turbulence. A detailed discussion of these waves is beyond the scope of the present paper but will be addressed in a subsequent publication.)

**Figure 3.**
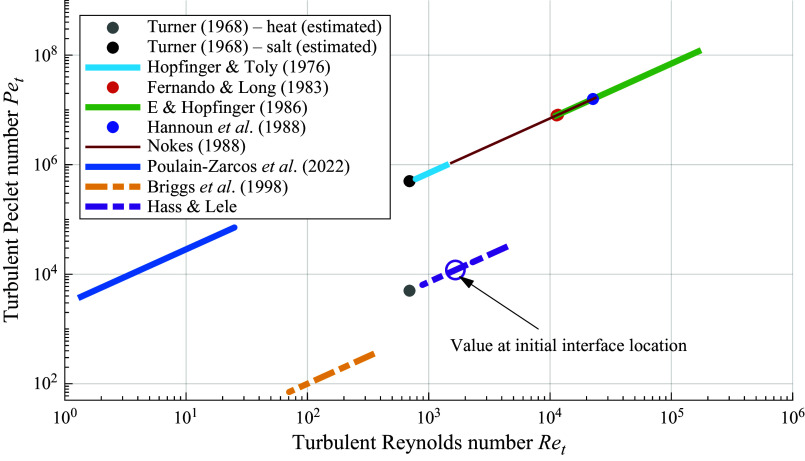
Figure 3 long description. Reynolds–Peclet number space traversed by a number of previous studies. Solid lines are used for OGT experiments and dashed lines for simulations. When values of the horizontal velocity scale u and integral length scale L were not reported in the references, the empirical correlation $uL=C\beta fS^{\left(3/2\right)}M^{\left(1/2\right)}$ was used, where f is the oscillating frequency of the grid, S the stroke length and M the mesh size. The empirical constants C=0.25 and β=0.1 were used unless otherwise specified in the reference. The purple open circle marks the value of ${\text{Re}}_{t}$ and ${Pe}_{t}$ at the initial location of the density interface in our simulations since, in our simulations, ${\text{Re}}_{t}$ is a function of depth (see § 4.1.2).

To contextualise the simulations within the larger body of shear-free turbulent studies, figure 3 shows where a variety of studies sit in the ${\text{Re}}_{t}$ – ${Pe}_{t}$ plane, where ${Pe}_{t}={\text{Re}}_{t}Pr$ is the turbulent Peclet number. The Reynolds number is roughly an order of magnitude larger than in the DNS of Briggs *et al.* (1996, 1998), and nearly two orders of magnitude larger in Peclet number. Nevertheless, our simulations are still at much lower Peclet numbers than that achieved in typical salt-stratified OGT experiments. Table 4 quantifies the Richardson number range spanned by a number of studies for comparison to our simulations.

## 4. Results

We first present results from the homogeneous fluid simulations in § 4.1 and then the stratified case in 4.2. For the remainder of the paper, plots of statistical quantities are truncated based on two criteria: (i) statistics must be averaged for at least two eddy turnover times (i.e. 2k(z)/ϵ(z) ), and (ii) the data record from the stratified cases must contain at least 2 % of the maximum potential enstrophy. The first of these sets a lower bound on the amount of time averaging that is deemed sufficient. The second was chosen so that irrotational fluid motions do not contaminate the statistics (negligible turbulence exists beyond the density interface for the low-Froude-number cases, but velocity fluctuations are non-zero). The latter criteria is in terms of Ertel’s potential vorticity Π≡ω⋅∇T because it is an effective diagnostic variable to identify the turbulent/non-turbulent interface (Watanabe *et al.* 2018). Though the second criteria was evaluated, in every case criteria (i) was the limiting factor and so all plots in this section are truncated to the region where sufficient time averaging was possible.

**Table 4.** Range of Richardson numbers reported in previous studies. The use of different definitions from study to study precludes a direct comparison between references. Here $u_{h}$ and $L_{h}$ are the RMS horizontal velocity and integral length scale measured in the homogeneous fluid at the depth of the density interface; $u_{s}$ is the RMS horizontal velocity measured in the stratified fluid. The 3/140 factor in the middle column converts $\Delta bH^{3}/(u_{h}L_{h}{)}^{2}$ to $\hat{Ri}$ defined in Fernando & Long (1983) and reported in figure 12 of that paper. Here $L_{E}$ is the Ellison length scale and $\langle \rangle_{I}$ a vertical average over the interface. Both of these are defined in § 4.2.6.

Reference	$\Delta bL_{h}/u_{h}^{2}$	$(3/140)\Delta bH^{3}/(u_{h}L_{h}{)}^{2}$	$\Delta b\langle L_{E}\rangle_{I}/\langle u_{s}^{2}\rangle_{I}$
Hopfinger & Toly (1976)	6.23–78.11	—	–
Xuequan & Hopfinger (1986)	4.64–259.1	—	—
Fernando & Long (1983)	—	56.1–814.2	—
Hannoun *et al.* (1988)	70–100	—	—
Briggs *et al.* (1998)	—	—	1.37–19.8
Hass & Lele	5.46–355.6	15.7–705.6	5.01–30.3

### 4.1. Homogeneous fluid

We systematically varied the kinetic energy and turbulence length scale by a factor of two (see $k_{\text{tgt}}$ and $\kappa_{\text{min}}$ in table 3) to determine the effect of these input parameters. For each configuration, a simulation was run in a homogeneous fluid. This section summarises the findings and compares with OGT experiments.

#### 4.1.1. Qualitative flow features

**Figure 4.**
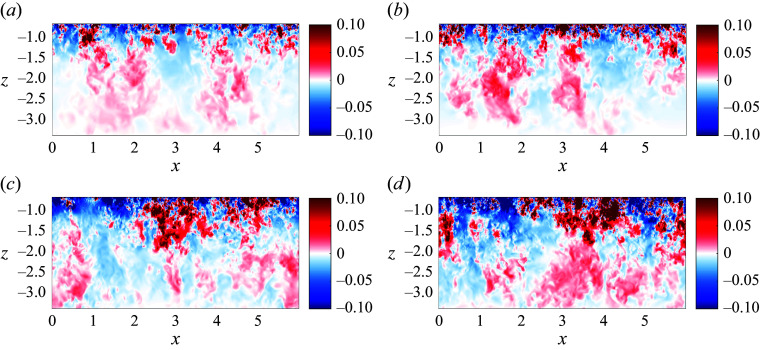
Figure 4 long description. Vertical velocity contours in the x – z plane. Results are shown for (*a*) SM71 ( $k_{\text{tgt}}=10$ , $\kappa_{\text{min}}=14$ ); (*b*) SM72 ( $k_{\text{tgt}}=20$ , $\kappa_{\text{min}}=14$ ); (*c*) SM73 ( $k_{\text{tgt}}=10$ , $\kappa_{\text{min}}=7$ ); (*d*) SM74 ( $k_{\text{tgt}}=20$ , $\kappa_{\text{min}}=7$ ).

**Figure 5.**
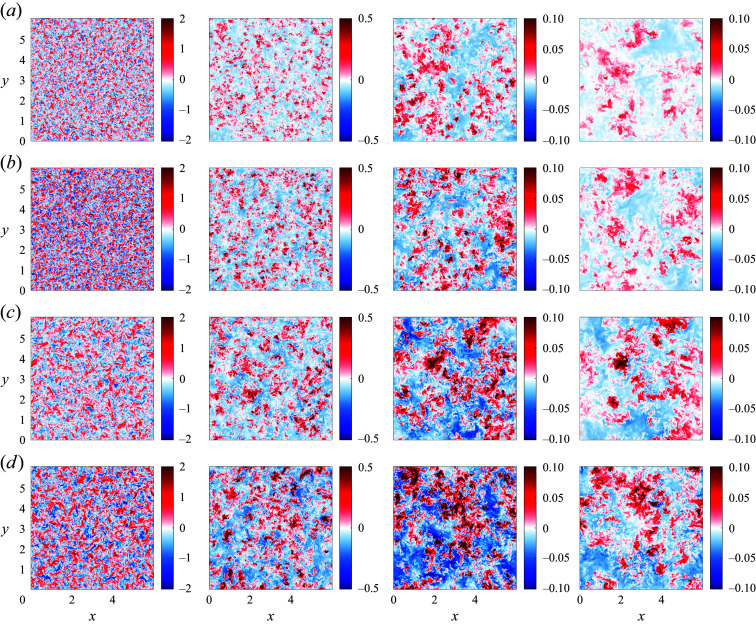
Figure 5 long description. The x – y planes of vertical velocity contours. Rows (top to bottom) show cases SM71, SM72, SM73 and SM74, respectively. Columns (left to right) correspond to depths $z^{\prime }=0.01$ , 0.76, 1.25 and 1.76. These depths can be compared with $l_{\;f}$ , the length scale based on kinetic energy and dissipation rate in the forcing region: $l_{\;f}≃0.20$ for SM71 and SM72, and $l_{\;f}≃0.34$ for SM73 and SM74.

Instantaneous vertical velocity contours are shown in figures 4 and 5. The influence of increasing the length and velocity scale in the forcing layer is visually striking and the high-Reynolds-number nature of SM74 is evident. (Case labels and associated parameters are summarised in table 3.)

#### 4.1.2. The TKE, dissipation rate and turbulence length scale

Thompson & Turner (1975) were the first to make measurements of the integral length scale $L_{\text{int}}$ and horizontal velocity scale u in OGT in a homogeneous fluid. Since then, multiple investigators have validated the general functional form of these on distance from the grid (Hopfinger & Toly 1976; Hannoun *et al.* 1988; Nokes 1988; Briggs *et al.* 1996; Poulain-Zarcos *et al.* 2022):

(4.1) $$\begin{array}{rl} u & =Au_{*}(z^{\prime }-z_{0}{)}^{-n}, \end{array}$$

(4.2) $$\begin{array}{rl} L_{\text{int}} & =\beta (z^{\prime }-z_{0}), \end{array}$$

where $z_{0}$ is a suitably defined virtual origin, $u_{*}$ is a velocity scale characterising the mechanical stirring, A is a constant with units of length raised to the power n and β is a non-dimensional constant. (Although (4.1) and (4.2) are typically reported in dimensional form in the experimental literature, here they are understood to have been non-dimensionalised using the scales introduced in § 2.1; hence, consistent with our convention, no asterisks are used.) Here $z^{\prime }$ is defined as positive downward (i.e. negative z ) consistent with the convention used in this paper. In oscillating grid experiments $u_{*}∼fS$ , where f is the frequency of oscillation and S is the stroke length (twice the amplitude) and A is a function of the grid geometry. (For example, $A=C_{1}M^{1/2}S^{3/2}$ is used in Poulain-Zarcos *et al.* (2022), where M is the grid mesh size and $C_{1}$ is a non-dimensional constant.)

The flow considered in this paper is statistically stationary, inhomogeneous in the vertical direction and possesses negligible mean velocity or shear. In such a setting, outside the region of forcing, the TKE equation (2.5*a*) reduces to

(4.3) $$\begin{array}{rl} \frac{dF}{dz} & =\epsilon . \end{array}$$

At a high enough Reynolds number, ϵ becomes independent of viscosity and scales with the large-scale turbulence, $\epsilon =Bu^{3}/L_{\text{int}}$ , where B is an order one constant. Given the equality in the TKE equation, this implies that $dF/dz∼u^{3}/L_{\text{int}}$ and so it is reasonable to assume that $F∼u^{3}$ , which was verified in our simulation data (not shown). Substitution of these scalings into (4.3) yields an ordinary differential equation for $u^{3}$ , i.e.

(4.4) $$\begin{array}{rl} \frac{du^{3}}{dz} & =B\frac{u^{3}}{L_{\text{int}}}, \end{array}$$

where the proportionality constant in $F∼u^{3}$ has been absorbed into B . It has been well established that in such a flow, the integral scale grows linearly with depth, i.e. $L_{\text{int}}=\beta z$ , leading to the solution

(4.5) $$\begin{array}{rl} u & =u_{r}(\frac{z}{z_{r}}{)}^{-B/3\beta } \end{array}$$

for $z\in [z_{r},\infty )$ , where $u_{r}=u(z_{r})$ is the velocity at the reference location $z_{r}>0$ . The origin for the coordinate frame describing the system was left unspecified in (4.4) and so, in general, we can write

(4.6) $$\begin{array}{rl} L_{\text{int}} & =\beta (z-z_{0}), \end{array}$$

(4.7) $$\begin{array}{rl} u & =u_{r}(\frac{z-z_{0}}{z_{r}-z_{0}}{)}^{-B/3\beta }\\ & =Au_{*}(z-z_{0}{)}^{-n}, \end{array}$$

(4.8) $$\begin{array}{rl} A & =\frac{u_{r}}{u_{*}}(z_{r}-z_{0}{)}^{n}, \end{array}$$

(4.9) $$\begin{array}{rl} n & =\frac{B}{3\beta }. \end{array}$$

This connects the empirical fits (4.1) and (4.2) to the governing equations.

The particular value of n observed in a given dataset is highly sensitive to the choice of virtual origin $z_{0}$ (Hopfinger & Toly 1976). We investigated reasonable choices for $z_{0}$ and their implications in Appendix C. We concluded from this study that the best choice for $z_{0}$ is defined by the equation $1/u(z_{0})=0$ , where $z_{0}$ is a weak function of the Reynolds number. This implicitly assumes that n=1 , which is verified *a posteriori* by its ability to collapse the data. Analogous to the experimental relationships above, we write, for our simulation data,

(4.10) $$\begin{array}{rl} \frac{k_{\text{fit}}}{k_{0}} & =A^{\prime }(\frac{z^{\prime }-z_{0}}{l_{0}}{)}^{-2n}, \end{array}$$

(4.11) $$\begin{array}{rl} l_{\text{fit}} & =\beta^{\prime }(z^{\prime }-z_{0})+l_{0}, \end{array}$$

where $k_{0}=k(z_{0})$ and $l_{0}=l(z_{0})$ are the kinetic energy and turbulence length scale at the virtual origin. Note that we are using $l=k^{3/2}/\epsilon$ as opposed to the integral of the two-point correlation (3.2) as is typically done in OGT experiments. We verified that l is proportional to the integral scale in our data (not shown here) but saw that the statistical variability in the integral scale was greater than in l , which was determined by looking at sub-records of each. For this reason, we use l in this section, but want to emphasise that none of our conclusions or scaling relationships change when using the integral scale. We used the label `fit’ to distinguish the algebraic expressions (4.10) and (4.11) from the LES TKE and length scale, the latter being used to define $k_{0}$ and $l_{0}$ .

By using l we are implicitly assuming a scaling relationship for ϵ , i.e.

(4.12) $$\begin{array}{rl} \frac{\epsilon_{\text{fit}}}{\epsilon_{0}} & =B^{\prime }(\frac{z^{\prime }-z_{0}}{l_{0}}{)}^{-(3n+1)}. \end{array}$$

No experimental analogue of this equation has been reported in the literature due to the difficulty in accurately measuring ϵ .

**Figure 6.**
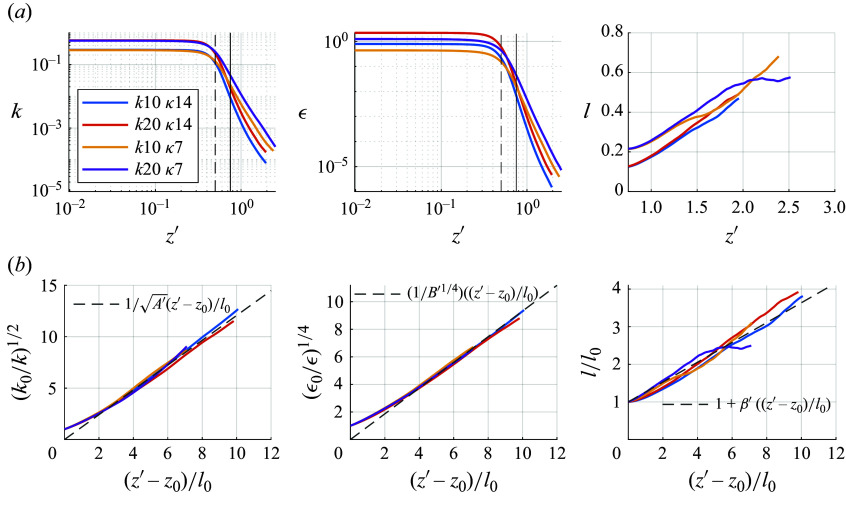
Figure 6 long description. (*a*) Unscaled TKE k , dissipation rate ϵ and turbulence length scale $l=k^{3/2}/\epsilon$ for all four unstratified cases; the legend reports $k_{\text{tgt}}$ and $\kappa_{\text{min}}$ for each case as ‘ k xx’ and ‘ κ xx’. (*b*) Scaled TKE, dissipation rate and length scale. Grey dashed lines are fits using the empirical coefficients $A^{\prime }=0.6867$ , $B^{\prime }=1.3221$ and $\beta^{\prime }=0.2636$ and the exponent n=1 (i.e. $k∼z^{\prime 2}$ and $\epsilon ∼z^{\prime 4}$ ). The figures in (*a*) show data inside the forcing layer. Data in (*b*) excludes this region since $z_{0}$ is outside the forcing layer.

The unscaled TKE, dissipation rate and turbulence length scale ( $l=k^{3/2}/\epsilon$ ) are plotted in figure 6(*a*). The power-law behaviour of k and ϵ and the linear growth of l are evident from these plots. By scaling the quantities according to (4.10)–(4.12) there is excellent collapse across all cases and we confirm the n=1 asymptotic power law by plotting $(k_{0}/k{)}^{1/2}$ and $(\epsilon_{0}/\epsilon {)}^{1/4}$ and observing a linear portion beginning around $(z^{\prime }-z_{0})/l_{0}=2.5$ (figure 6 *b*). The turbulence length scales also are much better represented by this scaling. The normalisation quantities $k_{0}$ , $l_{0}$ and $\epsilon_{0}$ are reported in table 5 for reference. The coefficients in (4.10)–(4.12) are inferred from the data. The individual curves from rearranging the algebraic expressions are shown in Hass (2025) and values are reported in the figure 6 caption. The lines resulting from these coefficients are overlayed as grey dashed lines in figure 6(*b*).

**Table 5.** Normalisation quantities used in empirical fits.

Run ID	$k_{0}$	$l_{0}$	$\epsilon_{0}$
SM71	0.0127	0.123	0.0116
SM72	0.0230	0.125	0.0278
SM73	0.0145	0.220	0.0080
SM74	0.0212	0.230	0.0133

Though $B^{\prime }$ in (4.12) was inferred from the data, the definition $\epsilon =u^{3}/l$ constrains its value, which gives a convenient sanity check on the empirical fits just derived. Using the definition of l , one can write

(4.13) $$\begin{array}{rl} \frac{\epsilon_{\text{fit}}}{\epsilon_{0}} & =\frac{k_{\text{fit}}^{3/2}}{\epsilon_{0}l_{\text{fit}}}\\ & =\frac{(k_{0}A^{\prime }{)}^{3/2}}{\epsilon_{0}}(\frac{\zeta }{l_{0}}{)}^{-3n}[\beta^{\prime }\zeta +l_{0}{]}^{-1}, \end{array}$$

where $\zeta \equiv z^{\prime }-z_{0}$ . Equating the right-hand-side of (4.13) to that of (4.12) yields an equation for $B^{\prime }$ :

(4.14) $$\begin{array}{rl} B^{\prime } & =\frac{\left(k_{0}A^{\prime }{)}^{3/2}(\zeta /l_{0}\right)}{\epsilon_{0}(\beta^{\prime }\zeta +l_{0})}. \end{array}$$

Asymptotically $\beta^{\prime }\zeta \gg l_{0}$ resulting in

(4.15) $$\begin{array}{rl} B^{\prime } & ≃\frac{A^{\prime 3/2}}{\beta^{\prime }}. \end{array}$$

Unfortunately, our computational domain is not large enough to test (4.15) as $\beta^{\prime }\zeta /l_{0}≲2.6$ .

**Figure 7.**
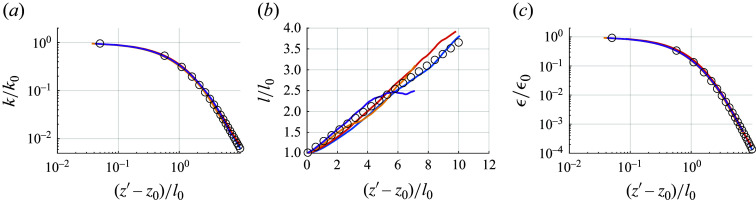
Figure 7 long description. Same scaling for k (*a*), l (*b*) and ϵ (*c*) as that in figure 6(*c*). From the smooth common profile of $k/k_{0}$ an empirical curve $k_{emp}$ is extracted (black circles). Similarly, a linear regression to the $l/l_{0}$ data yields a second empirical curve $l_{emp}$ (black circles). The black circles on the $\epsilon /\epsilon_{0}$ profiles (right figure) are obtained by plotting $k_{emp}^{3/2}/l_{emp}$ . Coloured profiles correspond to the legend in figure 6.

A more direct sanity check can be demonstrated by fitting a smooth curve to the $k/k_{0}$ profiles in figure 6(*c*) and a line to those of $l/l_{0}$ . These purely empirical fits can then be scaled to give units of $\epsilon /\epsilon_{0}$ and overlayed on the dissipation rate profiles. This process is illustrated in figure 7 where the black circles are the empirical fits. The asymptotic power law of $\epsilon /\epsilon_{0}$ is closely matched by such a procedure.

From the dissipation rate and kinetic energy, a Reynolds number can be defined:

(4.16) $$\begin{array}{rl} {\text{Re}}_{t} & =\text{Re}\frac{k^{2}}{\epsilon }. \end{array}$$

In terms of the scaling relationships above, this can be written as a function of $z^{\prime }$ showing that, for n=1 , the Reynolds number should be constant, i.e.

(4.17) $$\begin{array}{rl} \frac{{\text{Re}}_{t,\text{fit}}}{{\text{Re}}_{0}} & =\frac{k_{\text{fit}}^{2}\epsilon_{0}}{k_{0}^{2}\epsilon_{\text{fit}}} \end{array}$$

(4.18) $$\begin{array}{rl} & =\frac{{A^{\prime }}^{2}}{B^{\prime }}(\frac{z^{\prime }-z_{0}}{l_{0}}{)}^{1-n}. \end{array}$$

**Figure 8.**
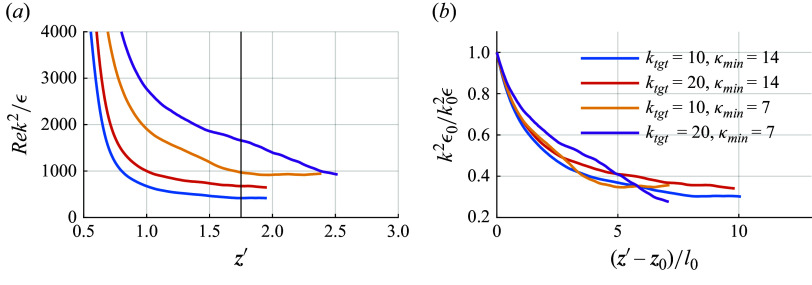
Figure 8 long description. Unscaled turbulent Reynolds number (*a*) and the Reynolds number normalised by the reference quantities at $z_{0}$ (*b*). The black vertical line in the left figure marks the initial density interface location for the stratified runs discussed below.

This is plotted in figure 8. All but the highest Reynolds number case (purple line) have a constant region. The departure could be due to insufficient time averaging, though we cannot say for certain.

Finally, we want to acknowledge that a more elaborate fit was proposed by Matsunaga *et al.* (1999) based on the analytical solution to the k - ϵ turbulence model equations that recovers the constant plateau at small values of ζ and an asymptotic power law for ζ≫1 . Such a fit matched the experimental data of Poulain-Zarcos *et al.* (2022) very well. We experimented with this form and found it did not agree with our data (Hass 2025) and preferenced the simpler (asymptotic) power-law forms (4.10)–(4.12) due to their connection to the governing equations and theoretical predictions of Long (1978*b*).

#### 4.1.3. Large-scale isotropy

One measure of large-scale isotropy is the ratio of vertical and horizontal root-mean-squared velocities, known as the isotropy factor $I\equiv w_{\text{rms}}/u_{\text{rms}}$ . Oscillating grid turbulence experiments report values in the range I∈[1,1.4] (Hopfinger & Toly 1976; Hopfinger & Linden 1982; Hannoun *et al.* 1988; De Silva & Fernando 1994; Kit, Strang & Fernando 1997). McCorquodale & Munro (2017) make a convincing case that reported values greater than one are due to contamination by the secondary flow generated by the sidewalls that is known to plague these experiments (McKenna & McGillis 2004). They suggest taking averages only in the centre of the tank where the mean flow is negligible and they show that when this is done, a value of I≃1 is achieved.

**Figure 9.**
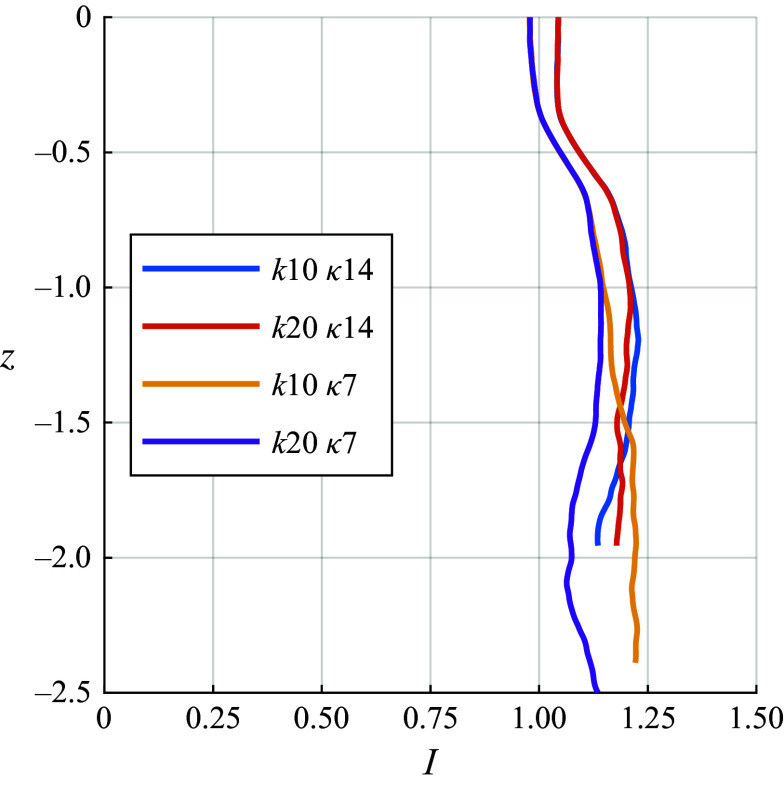
Figure 9 long description. Isotropy factor, $I\equiv w_{\text{rms}}/u_{\text{rms}}$ . See figure 6 caption for legend interpretation.

We found in our numerical experiments that while the turbulence is nearly isotropic in the forcing layer, it relaxes to a constant value of 1.125≲I≲1.25 (figure 9). This is within the range reported in experiments. The two other numerical studies of a similar problem that we know of report values of I=1.1 (Bodart, Cazalbou & Joly 2010) and I=1.4 (Briggs *et al.* 1996).

### 4.2. Stratified cases

**Figure 10.**
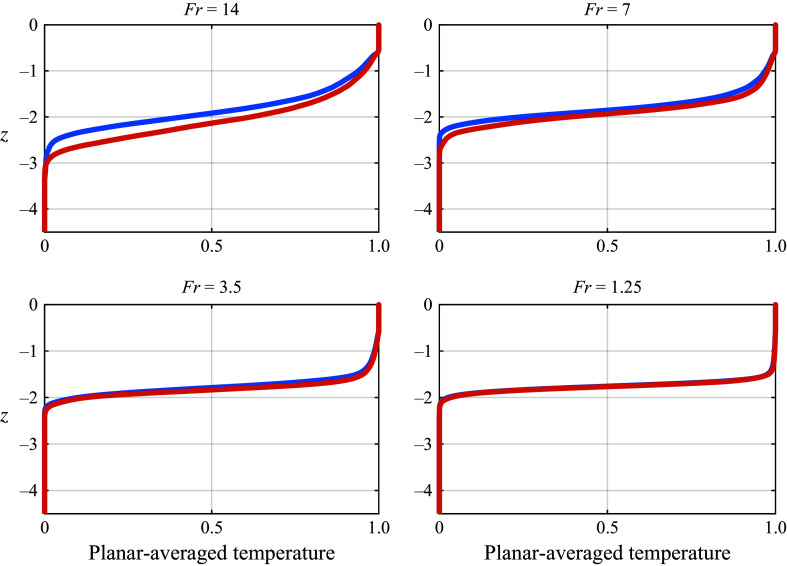
Figure 10 long description. Planar-averaged temperature profiles at the start and end of time averaging.

The notion of time averaging, mentioned in the beginning of this section, is an appropriate statistical mean for the unstratified cases since they are statistically stationary. However, time averaging in the stratified case is not strictly valid; the rate of work of the forcing layer on the fluid remains constant and, therefore, the density interface continues to move as turbulence entrains the heavier, non-turbulent fluid. A common approximation used in such situations, and used in our study, is to average over a brief window and argue that the flow is quasi-stationary. (The quasi-stationary approximation states that the turbulence time scale is much smaller than that associated with mean-state evolution.) Figure 10 shows the mean temperature profiles at the beginning and end of the averaging window for the four stratified cases. The elapsed time represented by these profiles is recorded in table 3. The quasi-stationary assumption is questionable for the weakly stratified case ( Fr=14 ), but a much better approximation for the other three. Averaged quantities reported in the remainder of the paper are planar and time averaged unless otherwise specified.

**Figure 11.**
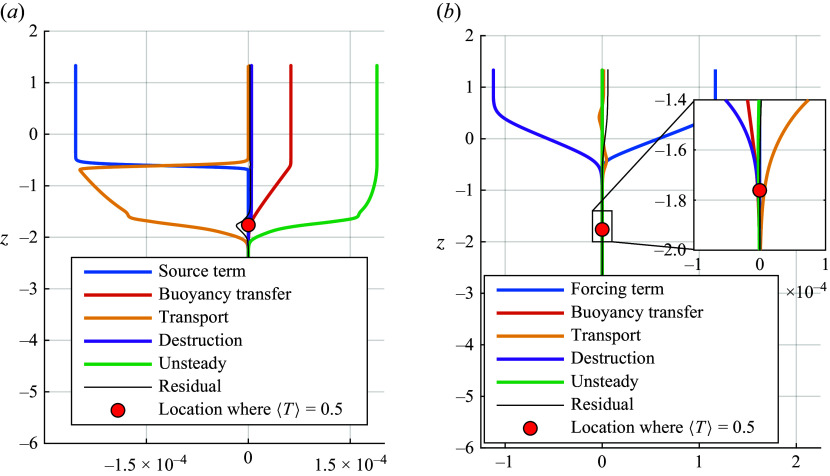
Figure 11 long description. Integrated energy budgets for case SM126 ( Fr=1.25 ). (*a*) Potential energy budget (2.9). (*b*) Kinetic energy (KE) budget (2.7). The other simulations have similar figures. The non-zero residual (thin black line) in the forcing region for kinetic energy is due to under-resolved dissipation. Red circles mark where ⟨T⟩=0.5 . The inset in (*b*) shows the KE balance in the interfacial region demonstrating that buoyancy transfer becomes a non-negligible sink in this area. The legend items are descriptive names for the terms in (2.7) and (2.9): Source/forcing term, $S_{P}$ / $F_{K}$ ; buoyancy transfer, B ; transport, $\Delta F_{P}$ and ΔF ; destruction, $\epsilon_{P}$ and $\epsilon_{K}$ ; unsteady, −∂P and $-\partial_{t}K$ .

#### 4.2.1. Flow characterisation – integrated energetics

The upper limit of integration in (2.7) and (2.9) is variable and so these budgets can be plotted as functions of depth (figure 11). If integrating over the full domain ( z=1.35 in the figures), we see that kinetic energy is balanced by the input from the forcing term and dissipation, and therefore, there is no (appreciable) net accumulation of kinetic energy in the domain. Conversely, the potential energy has a significant unsteady term resulting from the effective sink provided by the source term in the heat equation. By heating the fluid at the edge of the forcing layer, we are draining potential energy from the system. The rate at which potential energy is removed is not balanced by the turbulent buoyancy flux and, therefore, there is a net (negative) rate of change of the domain-integrated potential energy. If we restrict our integration to be below the forcing layer edge then the rate of change is explained by the significant transport out of the control volume.

The entrainment rate of the density interface is governed by the net effect of kinetic energy injection and potential energy removal (i.e. heating in the forcing layer). One can envision a scenario where the heat source is so significant that it completely arrests mixed-layer deepening despite a constant rate of kinetic energy injection. In our simulations the ratio of the potential energy removal rate to the kinetic energy injection rate, $S_{P}/F_{K}$ (see (2.7) and (2.9)), is at most $2.1\times 10^{-4}$ and so we conclude that the potential energy extracted from the domain has no discernible effect on the entrainment rate.

We end this discussion by pointing out that while the amount of relative energy removed to that injected is negligible, the contribution of $S_{T}$ to the buoyancy flux budget is not. The relative magnitude of $\int_{z}\langle w^{\prime }S_{T}^{\prime }\rangle dz$ to the maximum (absolute value) term in the $\left\langle w^{\prime }T^{\prime }\right\rangle$ budget is 16 %, 12.5 %, 4.8 % and 0.5 % for the cases with Fr=14 , 7, 3.5 and 1.25, respectively.

#### 4.2.2. Qualitative flow features

**Figure 12.**
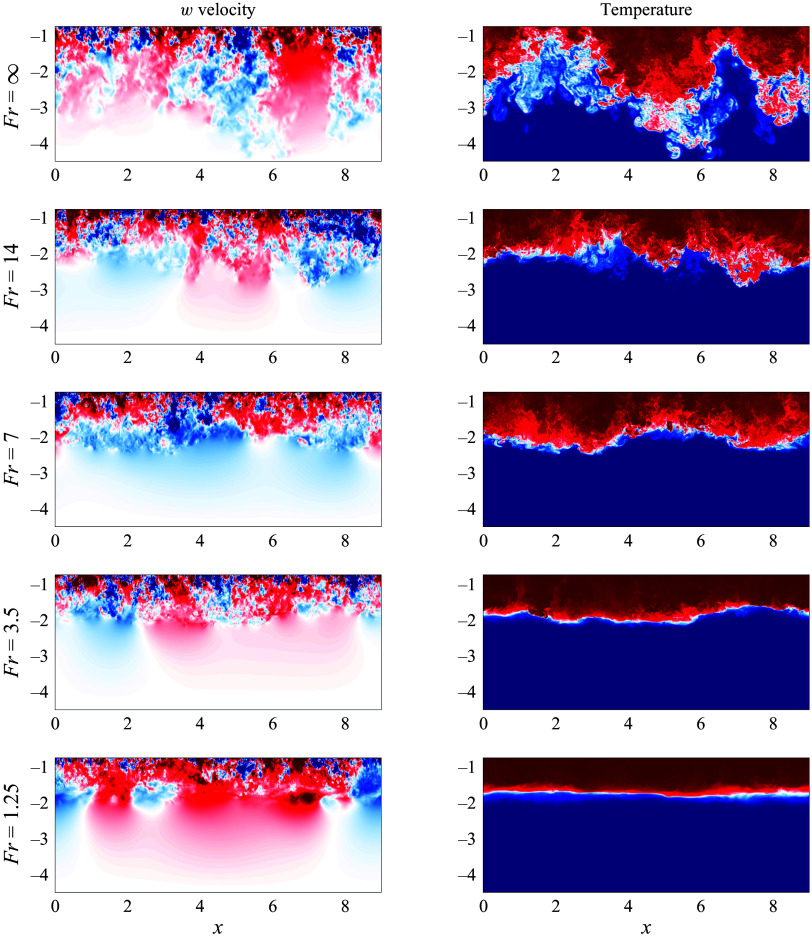
Figure 12 long description. Instantaneous contours of vertical velocity and temperature in the x – z plane at $y=L_{y}/2$ . Snapshots are taken at roughly the same simulation time. Only a selected portion of the computational domain is visualised that excludes the forcing region above. Recall that the full domain is z∈[−5.025,1.35] . The colour scale is blue-to-red for w∈[−0.1,0.1] and T∈[0,1] .

**Figure 13.**
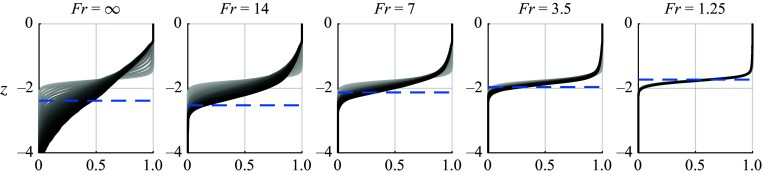
Figure 13 long description. Profiles of ⟨T⟩(z,t) for $t\in [t_{0},t_{\;f}]$ where the lines get darker as $t\to t_{\;f}$ . Blue dashed lines mark the location where the time window used for averaging is equal to two eddy turnover times (in the quasi-stationary state). This shows where time-averaged statistics are truncated in subsequent sections.

Figure 12 shows contours of vertical velocity and temperature for the stratified cases. The influence of increased stratification is clear from these figures where the turbulent eddies are flattened and the interface is significantly sharpened as Fr→0 . A quantitative characterisation of these effects will be provided in subsequent sections. Figure 13 shows the evolution of the mean temperature profile where the lines are coloured light to dark for early to late times, respectively. The rate of mixed-layer deepening is qualitatively evident from these plots, ranging from relatively rapid deepening for unstratified cases towards quite slow deepening for the Fr=1.25 case.

**Figure 14.**
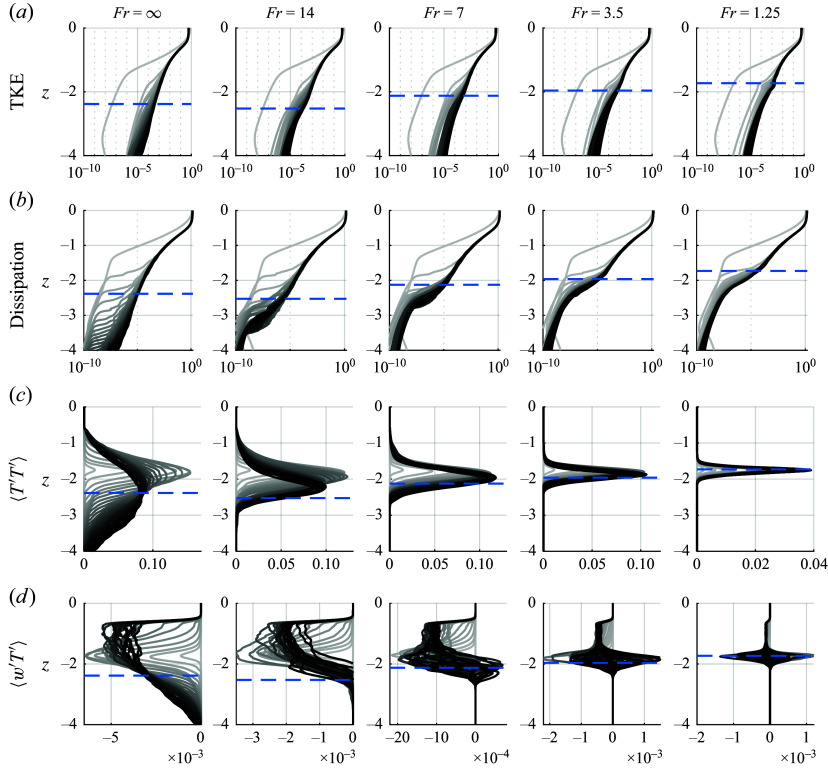
Figure 14 long description. Instantaneous profiles of the planar ( x – y ) averaged (*a*) TKE, (*b*) dissipation rate, (*c*) scalar variance and (*d*) scalar flux. Light coloured lines are at early times, which become progressively darker as time progresses. See figure 13 caption for an explanation of the blue dashed lines. Note the log scale used in (*a*) and (*b*).

Evolution from the initial condition to the quasi-stationary state is visualised in terms of TKE, TKE dissipation rate, scalar variance $\left\langle T^{\prime 2}\right\rangle$ and scalar flux $\left\langle w^{\prime }T^{\prime }\right\rangle$ in figure 14. Note the presence of interfacial waves manifesting as oscillations (in time) of $\left\langle w^{\prime }T^{\prime }\right\rangle$ at the interface. This is especially apparent for the Fr=3.5 and 1.25 cases.

#### 4.2.3. Are we simulating a ‘two-layer’ system?

The temperature initial condition is a smooth hyperbolic tangent profile to approximate a two-layer system so that finite difference operators remain finite. The `sharpness’ of the transition can be quantified in terms of the length scale of impinging eddies at early times: $\delta_{I}/l_{I}≃0.25$ , where $\delta_{I}$ is the interface thickness bounded by ⟨T⟩∈[0.1,0.9] and $l_{I}$ is the large eddy length scale at the initial interface location. So, as far as initial eddies are concerned, the numerically smooth initial condition appears relatively sharp to incident turbulence. At later times there is a quasi-equilibrium between the dense fluid entrained by turbulence at the interface and the heating term $S_{T}$ at the forcing layer edge. During this quasi-stationary state a mean stratification exists within the mixed layer as is evident in figure 13 and more clearly revealed later (e.g. in figure 19).

The focus of this paper is on the quasi-stationary state, and so the non-zero background stratification is dynamically relevant and allows us to define local (pointwise in z ) non-dimensional parameters. Typically, in experimental studies of OGT, the overall stability of the flow is quantified in terms of a Richardson number defined by the buoyancy difference across the interface, since the mean density gradient is assumed negligible in the mixed layer. Obviously this parameter is also computable from our data (see discussion of the entrainment rate in § 4.2.6), but we can also analyse the continuous distribution of quantities such as the turbulent Froude number.

#### 4.2.4. Outer and inner scaling of turbulence quantities

**Figure 15.**
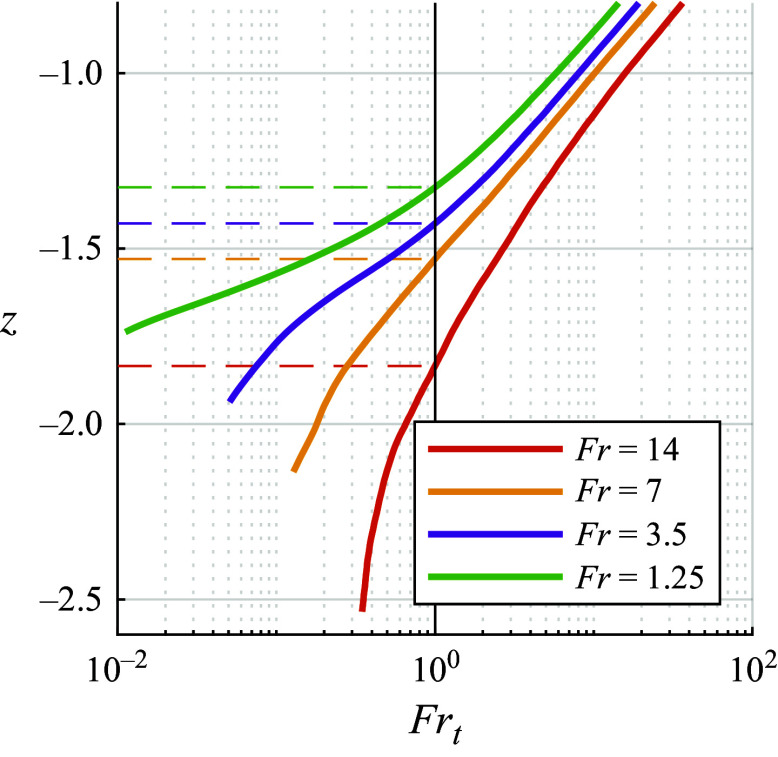
Figure 15 long description. Turbulent Froude number ${\text{Fr}}_{t}=\epsilon /Nk$ . The black vertical line marks ${\text{Fr}}_{t}=1$ and the horizontal dashed lines show where in the domain this value is reached for each simulation.

As turbulence diffuses from the forcing layer, it does not immediately encounter notable buoyancy effects and evolves as in an unstratified fluid. As the density interface is approached, buoyancy effects begin to play a dynamical role and modify the eddy structure and statistics. It is in this sense that we discuss ‘outer’ and ‘inner’ regions. Considering that in a high-Reynolds/Peclet-number flow a horizontal Froude number quantifies the ratio of inertial to buoyancy forces (Gargett 1988), we can gain intuition of where these regions exist by looking at the turbulent Froude number ${\text{Fr}}_{t}=\epsilon /Nk$ . (In the outer region the flow is locally isotropic and so the turbulent Froude number will convey the same information as a version computed from horizontal scales. We confirmed this, but do not show it here.) This is shown in figure 15.

**Figure 16.**
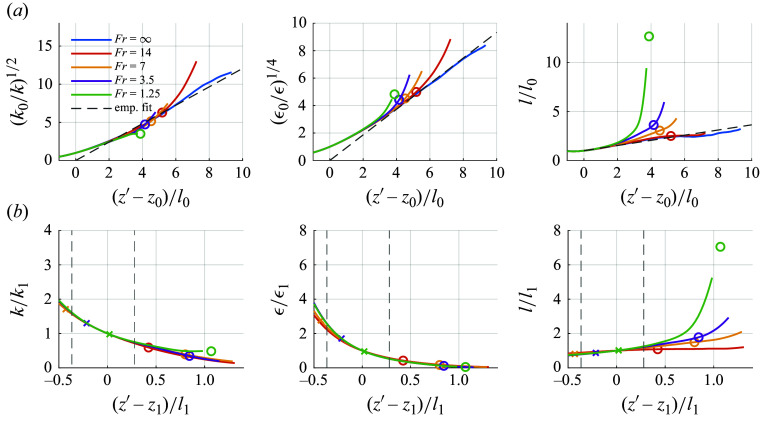
Figure 16 long description. Comparison of outer and inner scaling for kinetic energy, dissipation rate and length scale. (*a*) Outer scaling; the empirical fits of § 4.1.2 are shown as dashed grey lines, demonstrating the breakdown of the scaling due to buoyancy effects. (*b*) Inner scaling; the vertical dashed grey lines mark where the curves are within a half-standard deviation of each other. Coloured circles mark the location where ⟨T⟩=0.5 . Coloured `x’s mark where $(z^{\prime }-z_{0})/l_{0}=2$ , i.e. where outer-region scaling terminates.

Well above the location where ${\text{Fr}}_{t}=1$ the fluid density is comparable to what it is in the forcing region and, thus, buoyancy effects are negligible. The spatial distribution of turbulence quantities therefore remains close to their behaviour in unstratified conditions. This is shown in figure 16(*a*) where an empirical bound on this region is found to be the normalised distance $(z^{\prime }-z_{0})/l_{0}=2$ , i.e. approximately two eddy scales (note that we are plot the depth coordinate on the horizontal axis to be consistent with the scaling results presented for k and ϵ in § 4.1.2 - a convention only adopted for this section.) Beyond this distance buoyancy effects modify turbulence properties.

We term the neighbourhood around ${\text{Fr}}_{t}≃1$ the ‘inner region’. The outer scaling for k and ϵ begins to break down in this area as seen in figure 16(*a*). The spread of the curves, relative to the unstratified case, is quantified in table 6 at $(z^{\prime }-z_{0})/l_{0}=1$ and 2. Based on these metrics, it is reasonable to truncate the outer-region scaling at $(z^{\prime }-z_{0})/l_{0}=2$ .

We postulate that curves can be collapsed locally by shifting the origin to the ${\text{Fr}}_{t}=1$ location (denoted $z_{1}$ ) and scaling by k and ϵ there. Figure 16(*b*) demonstrates that a local collapse is achieved by such a rescaling.

**Table 6.** The spread of quantities in figure 16(*a*) relative to the unstratified simulation (blue curve in figure 16 *a*) at $(z^{\prime }-z_{0})/l_{0}=1$ and 2.

Quantity	$(z^{\prime }-z_{0})/l_{0}=1$	$(z^{\prime }-z_{0})/l_{0}=2$
$(k_{0}/k{)}^{1/2}$	2.02 %	4.37 %
$(\epsilon_{0}/\epsilon {)}^{1/4}$	0.95 %	1.33 %
$l/l_{0}$	3.63 %	15.9 %

**Figure 17.**
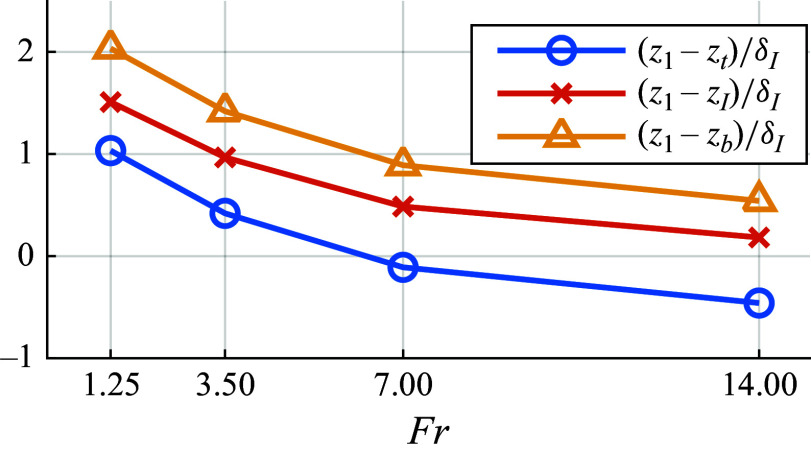
Figure 17 long description. Relative location of $z_{1}$ to the density interface. Here $z_{1}$ is defined by ${\text{Fr}}_{t}(z_{1})=1$ ; $z_{t}$ , $z_{I}$ and $z_{b}$ are defined in terms of the mean temperature profile: $\langle T\rangle (z_{t})=0.9$ , $\langle T\rangle (z_{I})=0.5$ and $\langle T\rangle (z_{b})=0.1$ ; $\delta_{I}$ is the interface thickness defined as $\delta_{I}=z_{t}-z_{b}$ .

The relative location of $z_{1}$ to the density interface is a function of the overall stratification. Shown in figure 17 is the distance between $z_{1}$ and the centre of the density interface (where ⟨T⟩=0.5 ) as well as the top and bottom of the interface (where ⟨T⟩=0.9 and 0.1, respectively). These distances are normalised by the density interface thickness $\delta_{I}$ . For the weakest stratification ( Fr=14 ), $z_{1}$ occurs almost at the centre of the interface indicating that turbulence is virtually unaffected by buoyancy until it is deep within the interfacial region. In this case, the primary entrainment mechanism is the large eddy engulfment of dense, non-turbulent fluid. At the other extreme, for Fr=1.25 , buoyancy effects become notable a full density interface thickness above the interface (which is $0.73k^{3/2}/\epsilon$ above the interface consistent with the notion that the kinematic blocking effect is limited to within one integral scale of the density interface; Hannoun *et al.* 1988). The result is that by the time eddies reach the interface, their vertical momentum has been so drastically reduced that the primary entrainment mechanisms are local shear instabilities and internal wave breaking. These are extremely rare events (Mcgrath *et al.* 1997) and so entrainment proceeds at a much reduced rate, which is quantified in § 4.2.6.

We note that the inner scaling for k and ϵ continues to work (i.e. successfully collapses the profiles) even if the local coordinate is defined by ${\text{Fr}}_{t}=0.5$ (not shown), but breaks down as we move to lower Froude numbers. Given the nature of our flow, very low Froude numbers are correlated with low Reynolds numbers and so viscosity becomes an important parameter in this region. Additionally, k is contaminated by internal gravity waves (i.e. sloshing interface) below this point.

**Figure 18.**
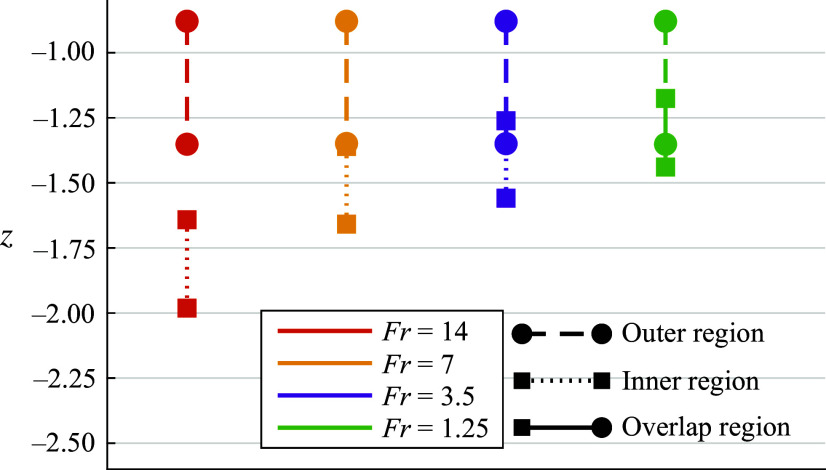
Figure 18 long description. Visualisation of the outer, inner and overlap regions referenced to unscaled depth for each case.

Vertical grey dashed lines drawn on figure 16(*b*) show where the inner scaling is valid. The bounds were determined empirically by requiring that the four curves have a coefficient of variation less than 7 % (the coefficient of variation is the mean-normalised standard deviation, also known as the relative standard deviation), which is the same coefficient of variation of the outer-scaled data at $(z^{\prime }-z_{0})/l_{0}=2$ , i.e. the empirically determined outer-region boundary. The two regions (outer and inner) are depicted in figure 18(*a*). The outer region is shown as circles connected by dashed lines whereas the inner region is bracketed by squares connected by dotted lines. A solid line identifies where the two regions overlap. It is interesting that the amount of overlap increases with stratification.

**Figure 19.**
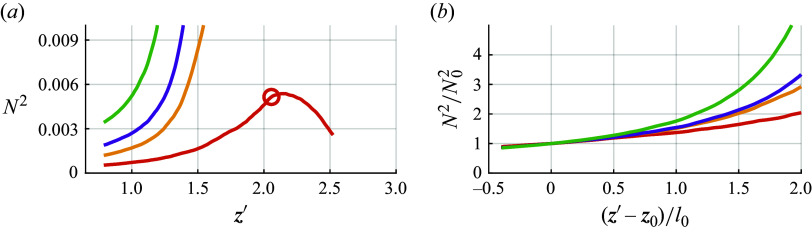
Figure 19 long description. $\text{The }N^{2}$ outer scaling. The open red circle marks where ⟨T⟩=0.5 for SM123. The ⟨T⟩=0.5 location for other cases is off the figure. The spread of normalised curves (*b*) at $(z^{\prime }-z_{0})/l_{0}=1$ is 25.0 % relative to the mean value at that location.

The common trend of ${\text{Fr}}_{t}$ in the outer region suggests that $N^{2}$ can also be collapsed by a simple scaling. Considering that turbulence quantities scale as in an unstratified fluid in the outer region, it is reasonable to assume buoyancy-related quantities scale with the ‘boundary condition’, i.e. their value at the virtual origin. Figure 19 demonstrates this scaling for $N^{2}$ , although the extent of the outer scaling, based on the coefficient of variation being no greater than 7 %, terminates at $(z^{\prime }-z_{0})/l_{0}=0.79$ . We verified a similar level of outer-region collapse for $\left\langle b^{\prime 2}\right\rangle$ and $\left\langle w^{\prime }b\prime \right\rangle$ using RMS values of b and w at the virtual origin (not shown here).

#### 4.2.5. Large-scale anisotropy

**Figure 20.**
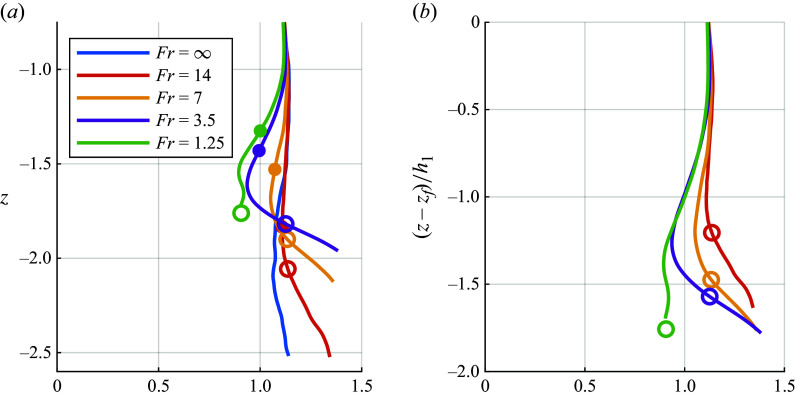
Figure 20 long description. Isotropy factor, $I\equiv w_{\text{rms}}/u_{\text{rms}}$ , for the stratified simulations. Open circles mark the location where ⟨T⟩=0.5 . Closed circles in (*a*) are where ${\text{Fr}}_{t}=1$ . The figures are the same, but the vertical axis in (*b*) is shifted to the forcing layer edge $z_{\;f}$ and normalised by the depth $h_{1}$ defined as $h_{1}=z_{\;f}-z_{1}$ , where $z_{1}$ is the location where the turbulent Froude number equals one, ${\text{Fr}}_{t}(z_{1})=1$ , i.e. the location of the closed circles in (*a*).

As the density interface is approached, we expect kinetic energy to be transferred from vertical motions to horizontal (see, for example, Hannoun *et al.* 1988). This is the trend we see initially in figure 20 where we have plotted the ratio of the vertical to horizontal RMS velocities (i.e. the isotropy factor I ). As the centre of the interface is approached, the moderate and strong stratification cases reverse course and the vertical RMS velocity is larger, which we interpret as interfacial waves contaminating the turbulence signal. Briggs *et al.* (1998) report similar behaviour in their simulations (see their figure 13) but only discuss the region in front of the interface where $w_{\text{rms}}/u_{\text{rms}}$ decreased.

Figure 20(*b*) shows the isotropy factor versus the scaled vertical coordinate $(z-z_{\;f})/h_{1}$ , where $z_{\;f}$ is the forcing layer edge and $h_{1}$ is the depth below $z_{\;f}$ where ${\text{Fr}}_{t}=1$ . This normalisation demonstrates that the upturn in the isotropy factor is below $h_{1}$ where ${\text{Fr}}_{t}$ is much smaller and where internal gravity waves will have a larger contribution to the dynamics.

We can also plot the distribution of the Reynolds stress anisotropy $b_{\text{ij}}$ defined as

(4.19) $$\begin{array}{r} b_{\text{ij}}=\frac{\left\langle u_{i}u_{\;j}\right\rangle }{2k}-\frac{1}{3}\delta_{\text{ij}}. \end{array}$$

**Figure 21.**
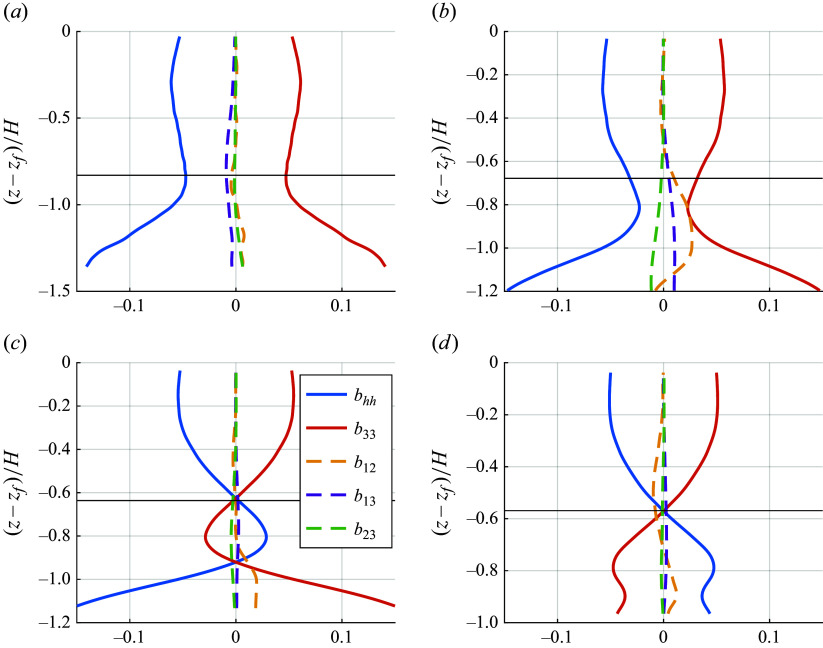
Figure 21 long description. Reynolds stress anisotropy. Depth is normalised by the height H defined as the distance below the forcing layer edge to the point where ⟨T⟩=0.5 . Horizontal black lines mark the location where ${\text{Fr}}_{t}=1$ . Results are shown for (*a*) *Fr* = 14 (SM123), (*b*) *Fr* = 7 (SM124), (*c*) *Fr* = 3.5 (SM125), (*d*) *Fr* = 1.25 (SM126).

Each component is plotted as a function of depth in figure 21(*a*–*d*) where $b_{hh}=b_{11}+b_{22}$ is the horizontal anisotropy. The depth coordinate is shifted to the forcing layer edge and normalised by the mixed-layer height $H=z_{\;f}-z_{I}$ . In general, the off-diagonal components are negligible compared with the horizontal and vertical components. We immediately notice that the location where the diagonal component $b_{33}$ minimises or crosses zero occurs in the neighbourhood of ${\text{Fr}}_{t}=1$ (marked by a black horizontal line in the figures), reiterating the dynamical significance of the Froude number. Based on these figures, where the horizontal and vertical anisotropies begin to converge some distance above the ${\text{Fr}}_{t}=1$ line, it appears the buoyancy-affected region may start before ${\text{Fr}}_{t}=1$ .

#### 4.2.6. Entrainment rate

**Figure 22.**
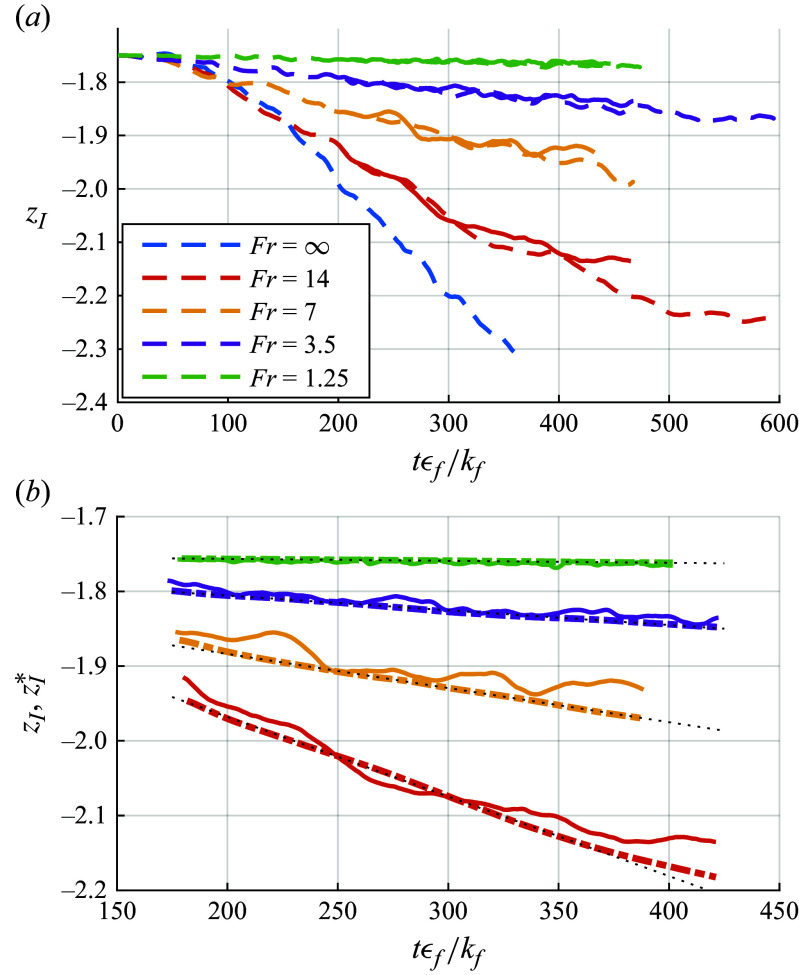
Figure 22 long description. (*a*) Evolution of interface location $z_{I}$ defined as $\langle T\rangle (z_{I})=0.5$ . Dashed lines are from the medium resolution cases and solid lines are the fine resolution runs. (*b*) Evolution of $z_{I}$ (solid lines) and $z_{I}^{*}$ (dash–dot lines) for the high-resolution stratified simulations. Black dotted lines approximate the linear portion of $z_{I}^{*}(t)$ . The time axis in (*b*) has been zoomed in on the high-resolution time record. Time is normalised in each figure by the eddy turnover time in the forcing region $k_{\;f}/\epsilon_{\;f}$ .

The relative deepening rate of each simulation is readily visualised by plotting the time evolution of the density interface location $z_{I}$ in figure 22(*a*). As pointed out by Nokes (1988), attempting to define an entrainment velocity from such a quantity via $dz_{I}/dt$ is difficult and imprecise due to the sloshing interface. He proposed to use the concentration of salinity in the mixed layer as a diagnostic for the amount of dense fluid that had been entrained. As discussed in § 4.2.3, the mean concentration (i.e. density) in the mixed layer is nearly uniform in OGT experiments, whereas in our simulation, due to the heating source, there is a mean gradient. However, we can still define an entrainment velocity from the evolution of the instantaneous Thorpe-sorted temperature profile $T^{*}$ , which is agnostic to waves (Thorpe 1977). This is because $T^{*}$ only changes due to irreversible mixing and not reversible stirring due to bulk fluid motions (Winters *et al.* 1995; Winters & D’Asaro 1996). Here $T^{*}$ is constructed by adiabatically resorting all fluid parcels (computational cells) into a statically stable profile (Winters *et al.* 1995; Peltier & Caulfield 2003). We define $z_{I}^{*}$ as the location where $T^{*}=0.5$ and overlay its evolution over that for $z_{I}$ for comparison in figure 22(*b*). The entrainment velocity can then be defined as

(4.20) $$\begin{array}{r} u_{e}=\frac{dz_{I}^{*}}{dt}. \end{array}$$

In practice, we fit a straight line through $z_{I}^{*}(t)$ and use the magnitude of the slope for $u_{e}$ . These fits are overlayed in figure 22(*b*) as black dotted lines. We note at late times for the Fr=14 case (red curve) that the entrainment rate appears to slow down. This is entirely expected as the incident turbulence has a different length and velocity scale than at the initial interface location. In OGT experiments the amount of deepening at the final time is much greater than in our simulations and so multiple entrainment rates can be measured in a single run (the slope of $z_{I}(t)$ changes in time/depth). Our simulations have not run long enough for this to manifest.

The entrainment hypothesis states that $u_{e}$ should be proportional to a characteristic velocity scale $u_{c}$ , i.e. $u_{e}∼Eu_{c}$ (Cenedese 2024). It has long been recognised that the ‘constant’ of proportionality E (known as the ‘entrainment rate’) is a function of the overall stability of the flow in a stratified environment. Molecular effects may also be important to the entrainment law (Turner 1968) and so, in general,

(4.21) $$\begin{array}{rl} u_{e} & =f(u_{c},L_{c},\Delta b,\nu ,\kappa ), \end{array}$$

where $u_{c}$ and $L_{c}$ are characteristic velocity and length scales of the turbulence, left unspecified for now. This expression can be non-dimensionalised as

(4.22) $$\begin{array}{rl} \frac{u_{e}}{u_{c}}=E & =g(\frac{\Delta bL_{c}}{u_{c}^{2}},\frac{u_{c}L_{c}}{\nu },\frac{u_{c}L_{c}}{\kappa }) \end{array}$$

(4.23) $$\begin{array}{rl} & =g(Ri,\text{Re},Pe). \end{array}$$

Experimental evidence suggests that the Reynolds number dependence goes away for moderately large Re , which is achieved in most experiments and almost certainly achieved in our simulations. It has been argued that the Pe dependence goes away for sufficiently large Pe . However, only Turner (1968) has varied Pe by changing κ and found different entrainment laws depending on κ . Hopfinger & Toly (1976) varied Pe by changing $u_{c}$ and $L_{c}$ and found that the results were independent of Pe for Peclet numbers sufficiently large.

In our simulations we cannot test the entrainment dependence on molecular diffusivity κ since all simulations utilise Pr=7.2 . Thus, we are interested in determining the functional dependence of E on Ri . In every case, a power-law relationship has been reported:

(4.24) $$\begin{array}{r} E∼{Ri}^{\gamma }. \end{array}$$

As discussed in the introduction, if the kinetic energy injected into the domain is proportional to the increase in potential energy due to mixing then γ=−1 (Turner & Kraus 1967; Long 1975). A lack of experimental agreement with this prediction prompted a number of more sophisticated theories yielding predictions of γ=−7/4 (Long 1978*a*), −3/2 (Linden 1973) and −5/3 (Fernando & Hunt 1997).

Typically, in OGT experiments the characteristic velocity and length scales are taken as the horizontal RMS velocity and horizontal integral scale in the homogeneous fluid ( $u_{h}$ and $L_{h}$ , respectively) at the depth of the density interface (where $z^{\prime }=H$ ). (When direct measurements are not taken, well-established correlations with experimental parameters are used. Such parameters include the oscillating frequency of the grid f , the mesh spacing M and the stroke length S .) These are unambiguous and enable a direct comparison between experiments. However, it is the turbulence in the vicinity of the density interface that is responsible for entrainment and so a more generally applicable parameterisation can be obtained by measuring these directly. Hopfinger & Toly (1976) attempted this and claimed that $u_{s}∼u_{h}$ , where $u_{s}$ is the RMS velocity in the stratified fluid, although their measurements did not extend closer than $1.5L_{h}$ to the density interface. Briggs *et al.* (1998), motivated to uncover a general parameterisation, postulated that the velocity and length scale of vertical motions near the interface should characterise the entrainment and proposed that

(4.25) $$\begin{array}{rl} \frac{u_{e}}{\langle w_{s}\rangle_{I}} & =f(\frac{\Delta b\langle L_{E}\rangle_{I}}{\langle u_{s}\rangle_{I}^{2}}), \end{array}$$

where $\langle \rangle_{I}$ is a vertical average over the interfacial region, $L_{E}=T_{\text{rms}}/(d\langle T\rangle /dz)$ is the Ellison length scale that characterises the vertical extent of overturning motions, and $u_{s}$ and $w_{s}$ are the horizontal and vertical RMS velocity in the stratified fluid. (It seems inconsistent to use a horizontal velocity scale when defining the Richardson number along with a vertical scale to normalise $u_{e}$ . As such, we only use a single velocity scale when plotting our entrainment data.) Given the various definitions used for $u_{c}$ and $L_{c}$ over the years we tabulate them for a subset of studies in table 7 along with the measured exponent γ in (4.24).

**Table 7.** $\text{Here }u_{h}$ and $L_{h}$ are the horizontal RMS velocity and integral length scale measured in a homogeneous fluid at the same depth as the density interface; $u_{s}$ and $w_{s}$ are respectively horizontal and vertical RMS velocities measured in the stratified fluid. The scale $L_{u}$ used in Turner (1968) is an unknown length scale that was held fixed since the grid parameters (mesh size and stroke length) were not varied. We denote by H the mixed-layer thickness. Xuequan & Hopfinger (1986) used well-established correlations between grid parameters and turbulence scales in a homogeneous fluid. Here S is the stroke length of the grid, f its oscillating frequency and M the distance between grid bars.

Study	$u_{c}$	$L_{c}$	γ
Briggs *et al.* (1998)	$\langle w_{s}\rangle_{I}$ and $\langle u_{s}\rangle_{I}$	$\langle L_{E}\rangle_{I}$	−1.5
Hopfinger & Toly (1976)	$u_{h}$	$L_{h}$	−1.5
Xuequan & Hopfinger (1986)	$0.3fS^{\left(3/2\right)}M^{\left(1/2\right)}H^{-1}$	0.1H	−1.5
Turner (1968)	$fL_{u}$	$L_{u}$	−1.5 (salt) and −1 (heat)
Nokes (1988)	fS	0.1H	−1.2±0.12
Fernando & Long (1983, 1985)	$u_{h}(L_{h}/H)$	H	−1.75

**Figure 23.**
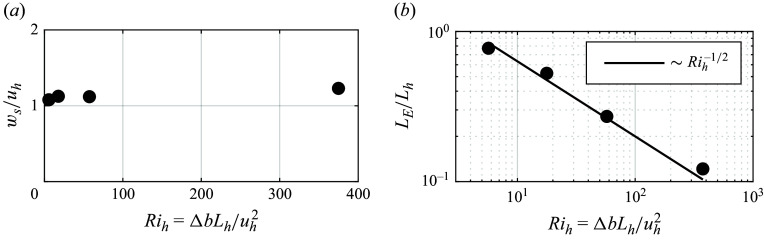
Figure 23 long description. Ratios of stratified and homogeneous fluid turbulence scales. (*a*) Ratio of velocity scales. (*b*) Length-scale ratios.

Briggs *et al.* (1998) report $E∼{Ri}^{-3/2}$ consistent with many high- Pe OGT experiments, which is surprising considering the relatively low Pe achieved in their DNS (see figure 3). It should be emphasised that the quantities used to normalise $u_{e}$ and define Ri in (4.25) are measured in the stratified fluid, unlike in experiments. To understand the differences, we plot the ratios $w_{s}/u_{h}$ and $L_{E}/L_{s}$ in figure 23 where, unlike Briggs *et al.* (1998), $w_{s}$ and $L_{E}$ are evaluated at the interface centreline (i.e. where ⟨T⟩=0.5 ) rather than averaged over the interfacial region. (The reason for this is simple: at high stratification the fluid below where ⟨T⟩=0.5 is highly intermittent and possibly not turbulent at all (see ${\text{Re}}_{b}$ in table 3) and so a vertical average obscures the true turbulent value.) We find that $w_{s}/u_{h}$ is independent of Ri and $L_{E}/L_{h}∼{Ri}^{-1/2}$ , in other words, the vertical length scale in the interface shrinks relative to the horizontal scale in the homogeneous fluid with increasing stratification.

To compare both to experiments and to Briggs *et al.* (1998), we define two entrainment rates and two Richardson numbers:

(4.26) $$\begin{array}{rl} E_{h} & =\frac{u_{e}}{u_{h}},\;\;\;\;{Ri}_{h}=\frac{\Delta bL_{h}}{u_{h}^{2}}; \end{array}$$

(4.27) $$\begin{array}{rl} E_{s} & =\frac{u_{e}}{w_{s}},\;\;\;\;{Ri}_{s}=\frac{\Delta bL_{E}}{w_{s}^{2}}. \end{array}$$

**Figure 24.**
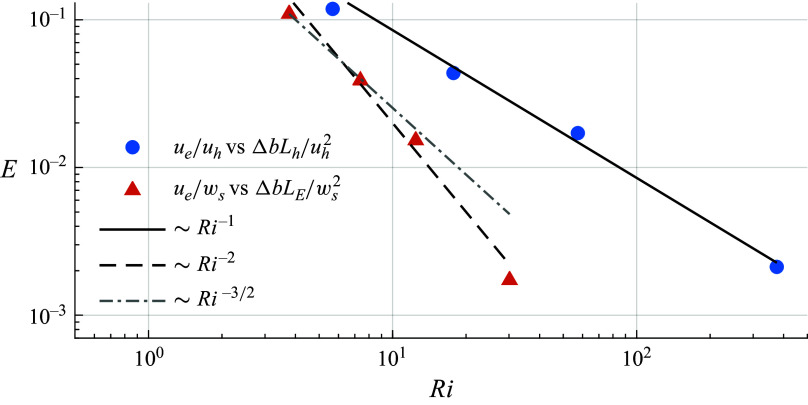
Figure 24 long description. Entrainment rate versus Richardson number. Two definitions of E and Ri have been used and two power laws observed: ${Ri}^{-1}$ and ${Ri}^{-2}$ . Blue circles use the RMS horizontal velocity and the integral scale (integral of the longitudinal autocorrelation function, (3.2)) in the homogeneous fluid. The homogeneous fluid quantities are measured at the depth of the density interface in the corresponding stratified simulation. Red triangles: the RMS vertical velocity and Ellison length scale measured where ⟨T⟩=0.5 in the stratified fluid.

Here, again, $w_{s}$ and $L_{E}$ are evaluated at the location where ⟨T⟩=0.5 . These are plotted in figure 24. There is a clear power-law relationship γ=−1 when using the homogeneous fluid scales, in agreement with the low- Pe experiment of Turner (1968). Such a scaling, coupled with the results of figure 23, suggest that $E_{s}∼{Ri}_{s}^{-2}$ , which matches the data quite well. We note that an exponent of −3/2 , as reported in Briggs *et al.* (1998), is not inconsistent with the three lowest stratification cases (grey dash–dot line in figure 24). Although the lowest-Froude-number case, SM126, departs from the −3/2 entrainment law and fallsmore rapidly, the following few cautionary notes are offered for consideration. First, SM126 does not resolve the Ozmidov scale in the interface (see figure 30) and so numerical artefacts could be in play; a higher resolution simulation would need to be run to say for certain. Second, the buoyancy Reynolds number in the interface is extremely low (see table 3) and so inferring a ‘turbulence’ scale at this location is questionable.

The observation of two different power laws for the entrainment rate in the same flow requires some additional comment. The scaling law using turbulence scales associated with the homogeneous fluid, (4.26), isolates the effect of buoyancy into a single parameter, ${Ri}_{h}$ , since $u_{h}$ and $L_{h}$ are both independent of stratification. This results in a clear separation between the inputs (homogeneous fluid turbulence properties $u_{h}$ , $L_{h}$ ) and the measure used to characterise the strength of stratification, ${Ri}_{h}$ , and the impacted output quantity, the entrainment rate $E_{h}$ . On the other hand, the use of interfacial scales $w_{s}$ and $L_{E}$ in scaling the stratification strength ${Ri}_{s}$ and entrainment rate $E_{s}$ (4.27) creates a more complex picture (effects of stratification are present in both the input and output). Viewed in this way, it seems more straightforward to use homogeneous fluid scales when answering the question: How does the entrainment rate depend on the overall stratification? We find a −1 power law for the entrainment rate dependence on the Richardson number ${Ri}_{h}$ . However, if one only has information about the interfacial turbulence, from field measurements for example, then a modified entrainment rate should be expected.

#### 4.2.7. Interface thickness

**Figure 25.**
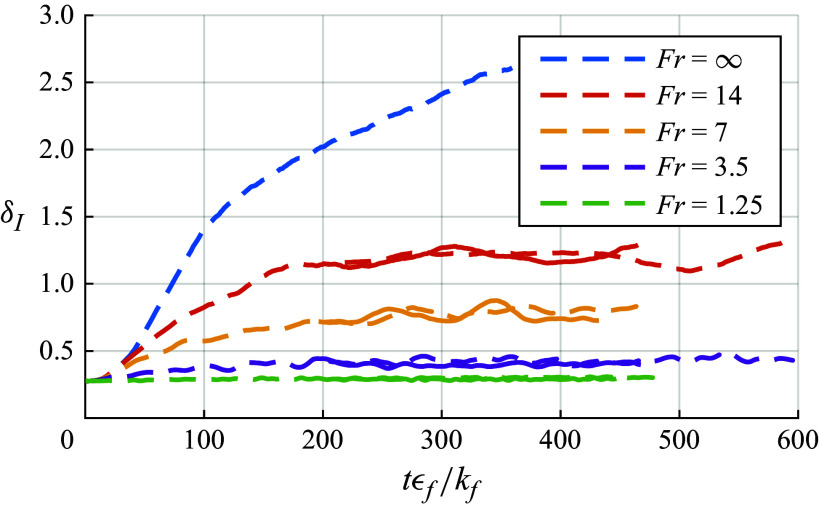
Figure 25 long description. Time evolution of the interface thickness. Solid and dashed lines have the same meaning as in figure 22.

**Figure 26.**
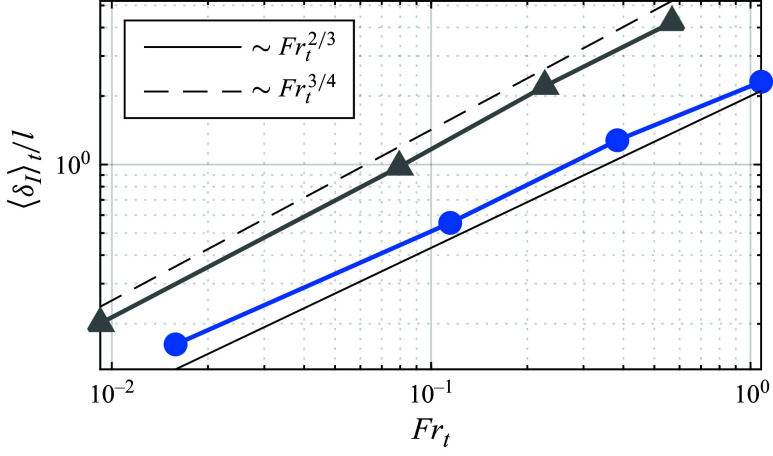
Figure 26 long description. Interface thickness versus turbulent Froude number. The interface thickness is time averaged over the fine-resolution time record (solid lines in figure 25). Blue circles use k and ϵ evaluated where ⟨T⟩=0.7 and grey triangles where ⟨T⟩=0.5 . The grey triangles have been multiplied by two to provide a clear presentation of both lines.

**Figure 30.**
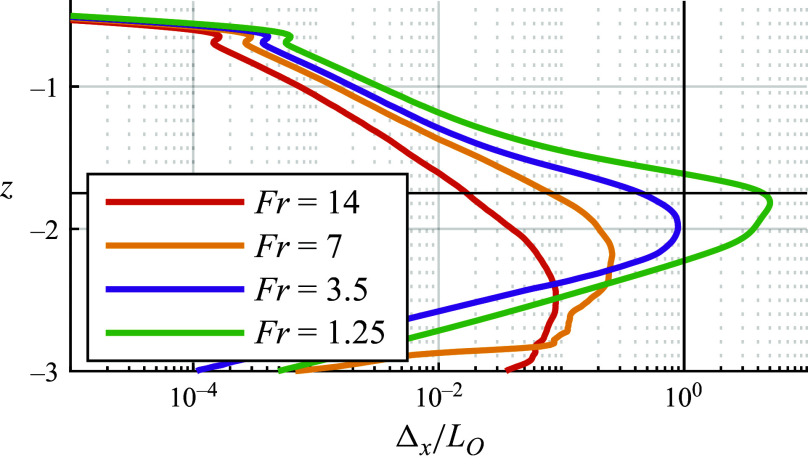
Figure 30 long description. Ozmidov scale resolution. The horizontal black line marks the initial density interface location and the vertical black line shows where LES should strive to be below in order to accurately measure mixing according to Khani (2018).

The significant sharpening of the interface as stratification increases is evident in figure 25 where $\delta_{I}$ is the interface thickness defined as the distance spanned by ⟨T⟩∈[0.1,0.9] . In each case, the interface thickens slightly from the initial condition and then appears to reach a constant equilibrium thickness. This is in contrast to the relationship $\delta_{I}∼H$ often reported in OGT experiments (see, e.g. Fernando & Hunt 1997). The thickness should be set by the turbulence parameters near the interface and the level of stratification, $\delta_{I}=f(k,\epsilon ,N)$ . Simple non-dimensionalisation of this expression yields

(4.28) $$\begin{array}{rl} \frac{\delta_{I}\epsilon }{k^{3/2}} & =g(\epsilon /kN) \end{array}$$

(4.29) $$\begin{array}{rl} \Rightarrow \frac{\delta_{I}}{l} & =g({\text{Fr}}_{t}), \end{array}$$

where $l=k^{3/2}/\epsilon$ defines the large eddy length scale. Plotting this quantity in figure 26 suggests that $\delta_{I}/l∼{Fr}_{t}^{\alpha }$ where 2/3⩽α⩽3/4 . This particular power law is sensitive to where the turbulence scales (used to normalise $\delta_{I}$ and compute ${\text{Fr}}_{t}$ ) are evaluated. The grey triangles are where ⟨T⟩=0.5 and the blue circles are where ⟨T⟩=0.7 .

The thickness $\delta_{I}$ , as defined, is a statistical thickness and does not represent the actual instantaneous thickness of the turbulent/non-turbulent interface, which presumably is of the order of the Batchelor scale (Watanabe *et al.* 2018). Looking at temperature contours in figure 12 gives an appreciation of how thin the interface is.

## 5. Discussion and conclusions

The simulations described and analysed in this paper are reminiscent of OGT experiments popular in the ‘60s–‘90s to study turbulent entrainment and mixing at a shear-free density interface. There are some notable differences mostly stemming from the potential energy sink term in our equations. However, this sink has negligible influence on the entrainment rate and so meaningful comparisons can be made. Furthermore, despite the differences, our scaling analysis is relevant to any system in which turbulence interacts with a sharp density interface.

The distinguishing feature of unstratified turbulence generated in a localised region of space is the self-similar decay of the velocity and growth of the length scale with distance from the source. When such turbulence is subject to a background stratification, this relationship is unmodified so long as ${\text{Fr}}_{t}\gg 1$ , which in our simulations corresponded to roughly two eddy scales from the forcing region. In the neighbourhood where ${\text{Fr}}_{t}≃1$ , turbulence quantities scale locally and collapse across a broad range of stratification strengths characterised by a global stability metric. Such an observation is a convenient diagnostic to appraise various turbulence closure schemes. For example, it has been shown that the k - ϵ Reynolds averaged Navier–Stokes model is analytically consistent with the observed velocity decay and length scale growth of OGT turbulence in an unstratified fluid (Matsunaga *et al.* 1999). However, in a stratified fluid, the buoyancy flux term in the k equation requires closure and it is not obvious that the prognostic fields k and ϵ would collapse in the neighbourhood of ${\text{Fr}}_{t}=1$ (not to mention the reliability of finding the ${\text{Fr}}_{t}=1$ location using such fields). By requiring that the model obey this fundamental result we can enable the modeller to make definitive choices about various model forms and optimally tune model coefficients. Furthermore, we noticed that there is an overlap between the inner and outer regions of the flow. The size of this overlap region increases with the overall stratification, an observation that will further aid in model validation.

Far beneath the point ${\text{Fr}}_{t}=1$ the situation becomes more complicated. For one, the combined effects of buoyancy and viscosity become important and the Reynolds number enters as an important parameter. We have not systematically studied such dual-parameter scalings due to limited data (only four cases have been studied) and, more importantly, the confounding influence of interfacial waves. This latter point is the second reason the region beneath ${\text{Fr}}_{t}=1$ is difficult to analyse unambiguously.

The ratio of vertical to horizontal RMS velocity, quantifying the large-scale aniostropy, followed the expected behaviour of decreasing as the density interface is approached, a clear signature of kinetic energy transferring from vertical to horizontal motions due to pressure-strain correlations. The effect of internal gravity waves is evident in this quantity as a reversal of the trend above the centre of the density interface.

The entrainment velocity, perhaps the single most important macro-scale quantity to predict, is, fortunately, agnostic to such waves and so can easily be measured. However, choosing an appropriate velocity scale to normalise the entrainment velocity is also sensitive to the presence of interfacial waves. Historically, researchers have proposed a universal functional dependence of the entrainment rate on a turbulence Richardson number. Quantifying this Richardson number also suffers from ambiguity in the presence of significant wave motion. Following most OGT experiments, we can avoid this ambiguity by utilising turbulence scales measured in the homogeneous fluid. When this is done, we found that the entrainment rate scales as ${Ri}^{-1}$ consistent with the low- Pe regime postulated by Turner (1968) and Hopfinger & Toly (1976). However, motivated to determine a general parameterisation connecting turbulence near the density interface to the entrainment, we used a similar definition of Ri as Briggs *et al.* (1998) based on vertical scales in the interfacial region and found that $E∼{Ri}^{-2}$ .

We also found the density interface thickness depended on the turbulent Froude number to a power between 2/3 and 3/4 , the precise value depending on where the data were measured. Previous authors have argued the interface thickness is independent of stratification (Crapper & Linden 1974; Long 1978*a*). On the other hand, Hannoun *et al.* (1988) reported a clear stratification dependence, and argued that the reason previous experiments did not measure this was due to insufficient observation techniques.

We hope that the present study will stimulate a greater computational effort to study the problem of shear-free entrainment as a fundamental component in the broader search for generalisable stratified turbulence models. The experimental database for this type of flow is quite extensive, but numerical simulations are limited to the present study and that of Briggs *et al.* (1996, 1998).

## Figures and Tables

**Figure 27.**
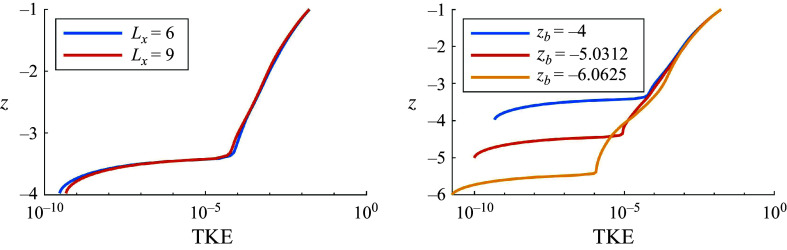
Figure 27 long description. Domain size sensitivity study. The precipitous drop of TKE in each case marks the edge of the sponge layer.

**Figure 28.**
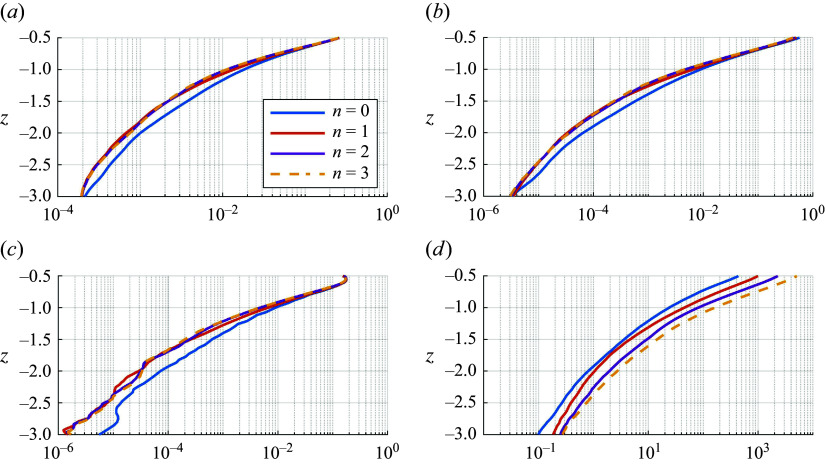
Figure 28 long description. Grid convergence study. (*a*) Turbulence kinetic energy. (*b*) Dissipation (viscous + SGS). (*c*) Transport. (*d*) Enstrophy.

**Figure 29.**
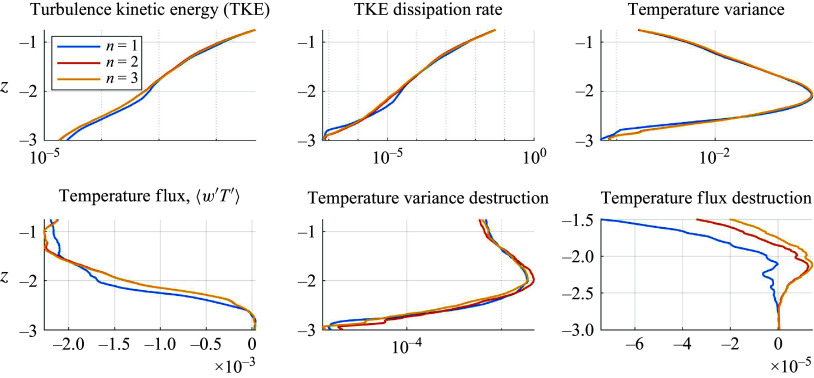
Figure 29 long description. Grid convergence study for the unstratified case SM123 ( Fr=14 ).

**Figure 31.**
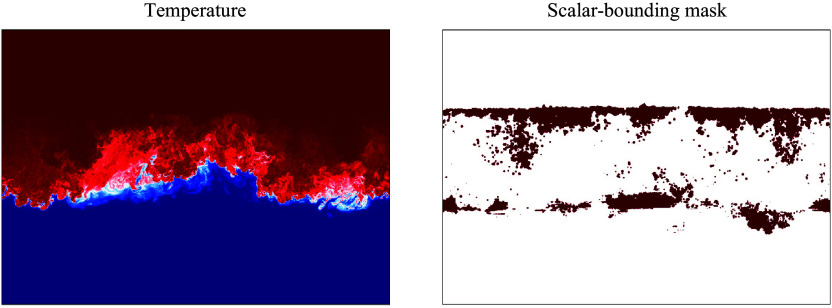
Figure 31 long description. Instantaneous contours of temperature and the mask used for the scalar-bounding model (i.e. red shows where the model is active and white is where the standard SGS model is used). This is for case SM123 ( Fr=14 ). The other cases are qualitatively similar.

**Figure 32.**
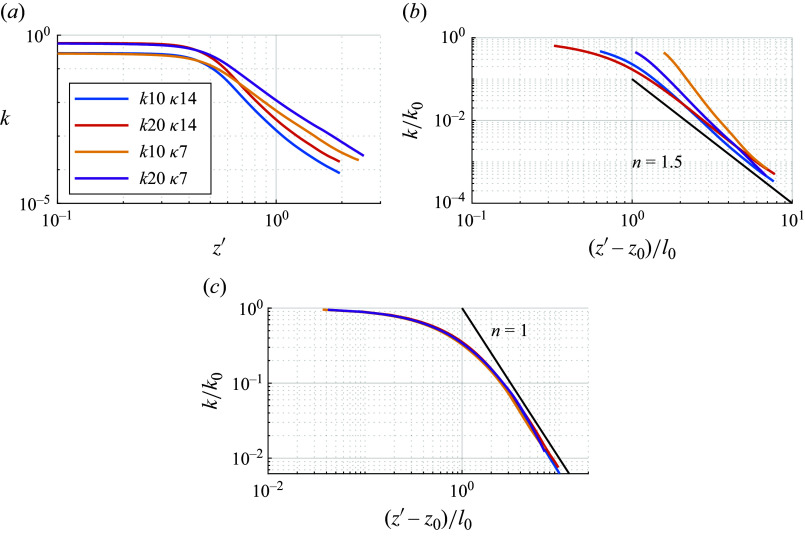
Figure 32 long description. The TKE profiles. (*a*) Unscaled (raw) data; as done previously, the legend reports $k_{\text{tgt}}$ and $\kappa_{\text{min}}$ for each case as ‘ k xx’ and ‘ κ xx’. (*b*) Scaled data referenced to $z_{0}$ determined from fitting a straight line to the l profiles. (*c*) Same as (*b*), but using $1/u_{\text{rms}}$ to determine $z_{0}$ . The black lines in the scaled TKE plots show power laws corresponding to −2n where n is reported on the figures.

**Figure 33.**
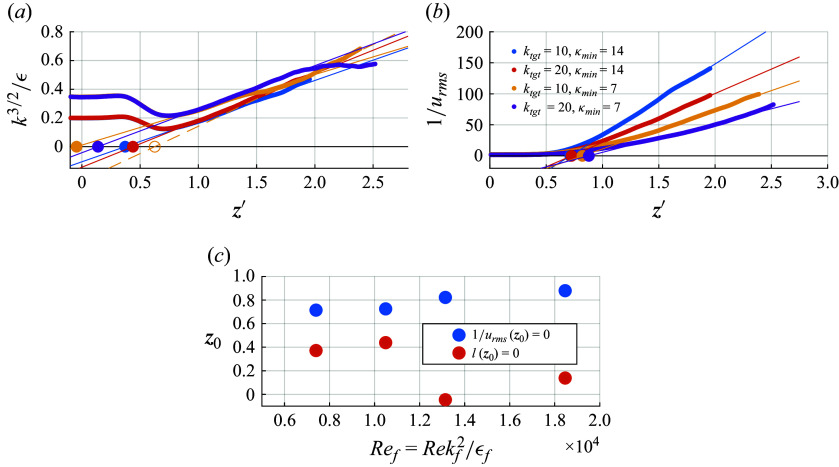
Figure 33 long description. Virtual origins computed from the turbulence length and velocity scales. (*a*) Profiles of the turbulence length scale l and the linear fits used in determining $z_{0}$ . (*b*) Profiles of $1/u_{\text{rms}}$ and the linear fits used in determining $z_{0}$ . (*c*) Variation of $z_{0}$ with forcing layer Reynolds number.

## Appendix A. Computational domain sensitivity studies

### A.1. Domain size sensitivity

For the unstratified case SM74 (highest Reynolds number), we ran a series of low-resolution simulations ( $\Delta_{i}=3/64$ ) varying the horizontal and vertical domain extents. The results of this study are quantified by TKE and shown in figure 27. There is not a significant change in the TKE profiles for larger domains and so we conclude that $L_{x}=L_{y}=6$ , $z_{b}=-4$ is sufficient for the unstratified runs. We used $L_{x}=L_{y}=9$ for the stratified simulations to build in a factor of safety, realising that horizontal length scales are amplified in these cases due to the ‘flattening’ of turbulent eddies by the density interface.

Sensitivity to the forcing-region size, its edge thickness and its proximity to the sponge layer was systematically investigated in Hass (2025). No significant sensitivity to computed statistics was observed by increasing these dimensions beyond that reported in table 2. See Hass (2025) for further details.

### A.2. Mesh resolution sensitivity

In order to test the sensitivity of various statistical quantities to mesh resolution we ran progressively finer meshes, refining by a factor of two in each dimension: $\Delta_{i,n}=3/(2^{n}\times 64)$ for n∈{0,1,2,3} . Figure 28 are from the high-Reynolds-number unstratified case (SM74) since this is the forcing configuration used in all stratified simulations. In order to standardise the comparison, statistics were averaged over the same length time record. Figure 28 shows the results for TKE, TKE dissipation rate (computed as the sum of resolved and modelled dissipation), TKE transport (sum of turbulent, pressure, viscous and SGS transport) and enstrophy. There is clear mesh sensitivity in the TKE and TKE budget terms up to n=2 with no notable change between n=2 and 3. While this suggests that the large scales are grid converged, the enstrophy plot demonstrates that the small scales are not, which is unsurprising since we are only beginning to approach the DNS regime for n=3 .

A similar grid convergence study was done for one stratified case, SM123 ( Fr=14 ), at three levels of mesh resolution: n∈{1,2,3} . The results of this study are shown in figure 29. Of the statistics reported in the figure, only the temperature flux destruction appear not to be grid converged over the entire depth. This term is defined as

(A1)
$$\begin{array}{rl} \epsilon_{wT} & =(\frac{1}{\text{Re}}+\frac{1}{\text{Re}Pr})\langle \frac{\partial T^{\prime }}{\partial x_{\;j}}\frac{\partial w^{\prime }}{\partial x_{\;j}}\rangle -\langle \tau_{3j}^{\text{SGS}}\frac{\partial T^{\prime }}{\partial x_{\;j}}\rangle -\langle q_{j}^{\text{SGS}}\frac{\partial w^{\prime }}{\partial x_{\;j}}\rangle . \end{array}$$

Further quantification of mesh resolution in terms of modelled and resolved dissipation/diffusion and molecular length scales is included in Hass (2025).

Finally, for stratified LES to give accurate predictions for mixing efficiency, Khani (2018) has shown convincingly that the maximum ratio of the grid spacing to the Ozmidov length scale $\Delta_{x}/L_{O}≃1$ . We plot this ratio for each case in figure 30, where we have not truncated the curves based on the statistical convergence criteria discussed in § 4.2. All cases are below the $\Delta_{x}/L_{O}=1$ cutoff except the lowest-Froude-number case (green curve) and for that case, only a small portion of the domain above the density interface (the initial interface location is shown as a horizontal black line).

## Appendix B. Scalar boundedness in LES

The transport of a scalar by a turbulent fluid is of interest in many geophysical and engineering applications. In addition to global conservation, the scalar must satisfy another physical constraint, namely boundedness that creates numerical modelling challenges. When solving the filtered Navier–Stokes equations, unphysical out-of-bounds excursions happen in the scalar field.

In order to guarantee boundedness, one must sacrifice solution accuracy. Various methods have been proposed in the literature including monotonicity-preserving limiting schemes (Suresh & Huynh 1997). We demonstrated in Hass (2025) that such schemes, while preserving the formal order of accuracy of the underlying numerical method, are overly diffusive. Conversely, the localised artificial diffusivity (LAD) scheme discussed in the following section results in a nearly bounded scalar and preserves the solution quality in regions that satisfy the physical constraints.

### B.1. Models

What follows is an abbreviated discussion of that provided in Hass (2025) and the reader is directed to that reference for further information. We take as the original model, the artificial diffusivity formulation proposed in Cook (2007). In the reference, the SGS model used to close the scalar-transport equation is augmented with an additional model diffusivity designed to target out-of-bounds scalar excursions. In Hass (2025), we proposed three modifications to the model in Cook (2007). Through a detailed evaluation of each we chose (B1):

(B1a)
$$\begin{array}{rl} \tilde{\kappa } & =[1-H(\kappa_{\text{SB}}-\kappa_{\text{th}})]\kappa_{\text{SGS}}+H(\kappa_{\text{SB}}-\kappa_{\text{th}})\kappa_{\text{SB}}, \end{array}$$

(B1b)
$$\begin{array}{rl} \kappa_{\text{SGS}} & =\frac{\nu_{\text{SGS}}}{Pr_{t}}, \end{array}$$

(B1c)
$$\begin{array}{rl} \kappa_{\text{SB}} & =C_{y}\bar{(\frac{\delta^{2}}{\Delta t_{CFL}}\eta )}, \end{array}$$

(B1d)
$$\begin{array}{rl} \delta & =\frac{\Delta_{i}|\partial_{i}T|}{\sqrt{\partial_{j}T\partial_{j}T}}, \end{array}$$

(B1e)
$$\begin{array}{rl} \eta & =|T|-1+|1-T|, \end{array}$$

(B1f)
$$\begin{array}{rl} \Delta t_{CFL} & =\min\{(|u|/\Delta_{x}+|v|/\Delta_{y}+|w|/\Delta_{z}{)}^{-1}\}, \end{array}$$

where $\nu_{\text{SGS}}$ is provided by an arbitrary SGS closure, in our case, the sigma model of Nicoud *et al.* (2011). Here $C_{y}$ is a user-tuned model constant. The overbar operator on $\delta^{2}\eta /\Delta t_{CFL}$ signifies the application of a spatial filter to smooth the model diffusivity field. In practice, we use a four-point Gaussian filter. Here H is the Heaviside step function.

Here $\tilde{\kappa }$ treats $\kappa_{\text{SGS}}$ and $\kappa_{\text{SB}}$ as mutually exclusive, by masking out $\kappa_{\text{SGS}}$ for $\kappa_{\text{SB}}>\kappa_{\text{th}}$ and *vice versa* for $\kappa_{\text{SB}}$ below this threshold value. This ensures only one model diffusivity coefficient is active in a given region of space in order to limit the amount of model diffusivity injected into the problem. This concept is similar to shock-capturing schemes in compressible flow simulations where only certain regions use the scheme based on a flow sensor and corresponding threshold.

### B.2. Model performance and parameter selection

To determine the optimal model settings, we primarily investigated three statistical quantities:

- the maximum and minimum scalar values at each instant in time,
- the probability density function of temperature,
- the volume fraction of the scalar-bounding mask H .

The first quantity shows the most egregious violation of the physical bounds whereas the second puts this in context of the entire volume. For example, if the maximum temperature exceeds the physical bound by 10 % say, but the probability of such excursions are exceedingly small, one may conclude this to be acceptable.

The final quantity, volume fraction of the scalar-bounding mask, is relevant because the LAD model is not a physics-based model, but a numerical treatment of numerical errors (unlike the SGS model). For this reason, we are interested in finding an optimal set of parameters that will limit the region in the domain where the LAD model is active to the greatest extent possible.

The full parameter sweep is reported in Hass (2025) and the final parameter choices are reported here:

(B2)
$$\begin{array}{r} C_{y}=100,\;\;\;\;\kappa_{\text{th}}=\frac{10}{Re\;Pr}. \end{array}$$

### B.3. Application of the model in the high-resolution simulations

The outcome of the parameter choice for $\tilde{\kappa }$ for the high-resolution simulations is documented here. The instantaneous domain-wide maximum and minimum temperature is reported in table 8. Included in the table are the probabilities of exceeding the physical bounds by more than 0.1 %, which is very low in all cases.

**Table 8.** Model performance in terms of maximum and minimum scalar excursions, probability of excursions and the volume fraction of where the model is active. The volume fraction is computed only in the region between the forcing layer edge and just beyond the density interface, i.e. the region where we expect $\tilde{\kappa }$ to be active. These values are for the SGS model used in the high-resolution simulations reported in the paper.

Case	$\max_{x,t}\{T\}$	$\min_{x,t}\{T\}$	P(T>1.001)	P(T<−0.001)	Mask volume fraction
SM123 (*Fr* = 14)	1.0055	−0.0026	2.959 $\times 10^{-6}$	1.1878 $\times 10^{-7}$	0.194
SM124 (*Fr* = 7)	1.0043	−0.0026	8.7269 $\times 10^{-7}$	3.0635 $\times 10^{-8}$	0.133
SM125 (*Fr* = 3.5)	1.0032	−0.0022	1.75 $\times 10^{-7}$	5.3993 $\times 10^{-10}$	0.087
SM126 (*Fr* = 1.25)	1.0023	−0.0004	1.6188 $\times 10^{-8}$	0	0.072

Additionally, for case SM123 ( Fr=14 ), we visualise the mask used to turn the scalar-bounding model on in figure 31. Also visualised are instantaneous temperature contours, which demonstrate that, unsurprisingly, the model is most active in the interfacial region where the mean density gradient and temperature fluctuations are maximum. The model is also active near the source region where temperature is forced to its maximum value and so the opportunity for out-of-bounds excursions is much more prevalent in this region. The time-averaged volume fraction for each case is also reported in table 8.

## Appendix C. An optimal definition of the virtual origin, $z_{0}$

As mentioned in § 4.1.2, the exponent n and coefficient β relating the velocity and length scales to the distance from the source region are very sensitive to the choice of virtual origin $z_{0}$ . The following three options have been proposed in the literature to determine its value.

- Extrapolate l to zero and $z_{0}$ marks the zero crossing (Thompson & Turner 1975; Hopfinger & Toly 1976; Poulain-Zarcos *et al.* 2022).
- Extrapolate 1/u to zero and $z_{0}$ marks the zero crossing (Hannoun *et al.* 1988; Briggs *et al.* 1996). (In effect, assuming *a priori* n=1 . However, only if n=1 is a reasonable value will data collapse across various cases.)
- Use the equilibrium position of the oscillating grid (Nokes 1988).

The latter choice is in light of the fact that the virtual origin computed from method (i) is often very close to this location, which has led some investigators to use this location without further verification. A systematic investigation of the virtual origin has not been reported in the literature, which motivated the inclusion of the present appendix. We computed virtual origins from our data based on definitions (i) and (ii) above. Not reported here, but included in Appendix C of Hass (2025), are results obtained by approximating definition (iii) based on the analogy between our source region and an oscillating grid. For each choice of virtual origin, we plotted the scaled TKE for each case in figure 32; see Hass (2025) for profiles of dissipation rate and turbulence scale. For comparison, the unscaled data are reproduced in figure 32(*a*).

We begin by determining $z_{0}$ from the linear (least-squares) fit of the length scale (definition i). The fits used are shown in figure 33(*a*). The domain over which the least-squares fit was calculated was determined by eye since both cases SM73 and SM74 (yellow and purple lines) have a plateau region that would greatly affect the fit if included. (Such a plateau in length scale z dependence has been reported in experiments (see, e.g. Poulain-Zarcos *et al.* 2022) so may be a typical feature in spatially decaying turbulence, though more time averaging would be required to say definitively.) Determining $z_{0}$ in this way varies significantly from case to case. Figure 33(*c*) shows $z_{0}$ as a function of the forcing-region Reynolds number ${\text{Re}}_{\;f}=\text{Re}k_{\;f}^{2}/\epsilon_{\;f}$ , where subscript f denotes a quantity averaged over the forcing layer. Here $z_{0}$ based on the l -fit method has a mean value of 0.2257 with standard deviation of 0.222 . Using $z_{0}$ from each case, we plot the scaled TKE in figure 32(*b*). Because the unscaled data demonstrate power-law decay, we view the lack of collapse as being due to the specification of $z_{0}$ and not the proposed model form. If instead we use the mean $z_{0}$ value from the four cases, we still do not achieve collapse of our profiles (Hass 2025). Because of this, we conclude that specifying $z_{0}$ based on the length scale l is not an optimal choice for our data. (This does not, however, invalidate computing $z_{0}$ this way in general. It is feasible that $z_{0}$ computed from l takes a much longer time to converge, something that is readily achievable in experiments, but not in our dataset.)

The virtual origin, $z_{0}$ , based on extending the linear portion of $1/u_{\text{rms}}$ (shown in figure 33 *b*) to zero has less variability across cases (figure 33 *c*), suggesting that it is a more robust approximation of $z_{0}$ than the l -fit method. Using four independent estimates of $z_{0}$ provides excellent collapse across all cases (figure 32 *c*). If instead we use the average $z_{0}$ value from the four cases, the collapse is not nearly as good (not shown here; see Hass 2025). The power law predicted from this method is n=1 and both the kinetic energy and dissipation rate figures show excellent agreement with −2n and −3n−1 , respectively (again, see Hass 2025 for the dissipation rate profiles).

Based on the figures above, and a more detailed investigation in Hass (2025), it is our recommendation that the virtual origin be determined from the plot of $1/u_{\text{rms}}$ given its superior ability to represent statistical profiles over a range of turbulence conditions.

## Table 1. Long description

A table listing power-law exponents from various studies. The table has two columns: Study and n. The Study column lists the names and years of different studies, while the n column lists the reported exponents. The studies include Thompson & Turner 1975 with an exponent of 1.5, Hopfinger & Toly 1976 with exponents ranging from 1 to 1.25, Hannoun et al. 1988 with an exponent of 1, Nokes 1988 with exponents ranging from 0.8 to 1.5, and De Silva & Fernando 1992 with an exponent of 1.

Navigate back to Table 1..

## Figure 1. Long description

The schematic illustrates a computational domain with a vertical axis labeled z, where z is defined positive downward. The domain includes a forcing region and numerical sponges at the top and bottom. The horizontal axes are labeled x and y. The domain features a varying cross-sectional area with specific heights labeled z1 and z2. The temperature profile at the initial time t equals zero is given by the equation T(t equals 0, z) equals 1 divided by 2 times the quantity 1 plus the hyperbolic tangent of the quantity z minus zi divided by d. The lengths Lf and Lsp are indicated for the forcing region and numerical sponges, respectively. The horizontal dimensions Lx and Ly are also shown.

Navigate back to Figure 1..

## Figure 2. Long description

The image contains two graphs. The first graph on the left shows the forcing layer mask function, with the x-axis labeled as g(z) and the y-axis labeled as z. The graph displays a red line that starts at the bottom left corner and quickly rises to a horizontal line near the top. The second graph on the right illustrates the absolute value of the dominant terms in the TKE budget. The x-axis is on a logarithmic scale, and the y-axis is labeled as z. The graph includes three lines: a blue line representing transport, an orange line representing dissipation, and a yellow line representing production due to forcing. The black dashdot line indicates the negligible TKE production due to forcing below a certain point. Thin black lines and dashed black lines mark the density interface and the edge of the forcing region, respectively.

Navigate back to Figure 2..

## Figure 3. Long description

A scatter plot illustrates the relationship between turbulent Peclet number and turbulent Reynolds number across multiple studies. The x-axis represents the turbulent Reynolds number, ranging from 10^0^ to 10^6^, while the y-axis represents the turbulent Peclet number, ranging from 10^2^ to 10^8^. The plot includes several data points from different studies, represented by various colors and line styles. Solid lines indicate OGT experiments, and dashed lines indicate simulations. Notable studies include Turner 1968, Hopfinger and Toly 1976, Fernando and Long 1983, E and Hopfinger 1986, Hannoun et al. 1988, Nokes 1988, Poulain-Zarcos et al. 2022, Briggs et al. 1998, and Hass and Lele. The purple open circle marks the value at the initial location of the density interface in the simulations. The plot shows a general trend of increasing turbulent Peclet number with increasing turbulent Reynolds number, with some variations and clusters among different studies.

Navigate back to Figure 3..

## Figure 4. Long description

A heat map displays vertical velocity contours in a plane, divided into four subplots labeled (a) to (d). Each subplot represents different experimental conditions. The x-axis ranges from 0 to 5, and the z-axis ranges from −3 to −1. The color scale on the right indicates values from −0.10 to 0.10, with blue representing lower values and red representing higher values. Subplot (a) shows a mix of red and blue regions with a slight concentration of red at the top. Subplot (b) exhibits a similar pattern but with more dispersed red regions. Subplot (c) has a more balanced distribution of red and blue areas. Subplot (d) shows a higher concentration of red regions, particularly at the top and middle sections.

Navigate back to Figure 4..

## Figure 5. Long description

A heat map displays vertical velocity contours for four cases labeled SM71, SM72, SM73, and SM74. The map is organized into four rows, each representing a different case, and four columns corresponding to depths of 0.76, 1.25, and 1.76. The color scale ranges from blue to red, indicating varying levels of vertical velocity. Blue areas represent lower values, while red areas indicate higher values. Each subplot shows a distinct pattern of velocity distribution, with some regions exhibiting higher intensity. The overall structure reveals how vertical velocity changes across different depths and cases, providing insights into the fluid dynamics of the system.

Navigate back to Figure 5..

## Figure 6. Long description

The image contains six graphs arranged in two rows. The top row shows three line graphs depicting unscaled turbulent kinetic energy, dissipation rate, and turbulence length scale for four unstratified cases. The legend indicates different values of k and kappa for each case. The bottom row shows three line graphs depicting scaled turbulent kinetic energy, dissipation rate, and length scale. Grey dashed lines represent fits using empirical coefficients and exponents. The figures in the top row include data inside the forcing layer, while the bottom row excludes this region. The graphs compare different cases and highlight the behavior of turbulence parameters.

Navigate back to Figure 6..

## Figure 7. Long description

The image contains three line graphs labeled (a), (b), and (c), each showing empirical data and linear regression curves. Graph (a) plots k/k0 against (z’-z0)/l0, graph (b) plots l/l0 against (z’-z0)/l0, and graph (c) plots ε/ε0 against (z’-z0)/l0. The black circles represent empirical data points extracted from a smooth common profile and a linear regression. Colored profiles correspond to the legend in figure 6. The x-axes are scaled similarly across all three graphs, and the y-axes represent different non-dimensional parameters. The graphs illustrate the relationship between these parameters and the length-scale ratio.

Navigate back to Figure 7..

## Figure 8. Long description

The image contains two line graphs. The first graph on the left shows the unscaled turbulent Reynolds number plotted against a variable labeled z prime. Four distinct curves are shown in different colors: blue, orange, yellow, and purple. The black vertical line marks the initial density interface location for stratified runs. The second graph on the right displays the Reynolds number normalized by reference quantities, plotted against a variable labeled z prime minus z0 over l0. The same four curves in blue, orange, yellow, and purple are present, each representing different parameter sets. The blue and orange curves correspond to k tgt equals 10 and 20 with kappa min equals 14, while the yellow and purple curves correspond to k tgt equals 10 and 20 with kappa min equals 7. All values are approximated.

Navigate back to Figure 8..

## Figure 9. Long description

A line graph displays the relationship between the variables I and z. The x-axis represents the variable I, ranging from 0 to 1.50, while the y-axis represents the variable z, ranging from 0 to −2.5. Four distinct data lines are plotted: a blue line labeled k 10 k 14, an orange line labeled k 20 k 14, a yellow line labeled k 10 k 7, and a purple line labeled k 20 k 7. Each line shows a downward trend as I increases. All values are approximated.

Navigate back to Figure 9..

## Figure 10. Long description

The image contains four line graphs, each representing planar-averaged temperature profiles at different Froude numbers. The graphs are arranged in a 2x2 grid. The x-axis represents the planar-averaged temperature, ranging from 0 to 1, while the y-axis represents the vertical position z, ranging from −4 to 0. Each graph shows two lines, one in blue and one in orange, indicating temperature profiles at the start and end of time averaging. The top-left graph corresponds to a Froude number of 14, the top-right to 7, the bottom-left to 3.5, and the bottom-right to 1.25. The temperature profiles show a consistent trend of increasing temperature with height, with the orange line generally indicating higher temperatures than the blue line at the same vertical position.

Navigate back to Figure 10..

## Figure 11. Long description

Two line graphs compare energy budgets for potential and kinetic energy in case SM126. The first graph (a) shows the potential energy budget with various terms such as source term, buoyancy transfer, transport, destruction, unsteady, and residual. The second graph (b) illustrates the kinetic energy budget with similar terms. Red circles mark locations where the average temperature is 0.5. The inset in graph (b) zooms in on the interfacial region, highlighting that buoyancy transfer becomes a significant energy sink in this area. The legend identifies each term’s descriptive name.

Navigate back to Figure 11..

## Figure 12. Long description

The image contains two sets of graphs, each with five subplots. The left set shows vertical velocity contours, while the right set displays temperature contours. Each subplot corresponds to a different Froude number, ranging from infinity to 1.25. The x-axis represents the horizontal distance from 0 to 8, and the y-axis represents the vertical distance from −4 to −1. The color scale transitions from blue to red, indicating variations in vertical velocity and temperature. The contours illustrate the distribution and interaction of these variables within the specified computational domain, excluding the forcing region above. The visual representation highlights the dynamic behavior of vertical velocity and temperature at different Froude numbers.

Navigate back to Figure 12..

## Figure 13. Long description

Five line graphs display profiles where the lines darken as they progress. Each graph represents different values of the Froude number, labeled as infinity, 14, 7, 3.5, and 1.25. The x-axis ranges from 0 to 1, and the y-axis ranges from −4 to 0. Blue dashed lines mark the location where the time window used for averaging is equal to two eddy turnover times in the quasi-stationary state. These lines indicate where time-averaged statistics are truncated in subsequent sections. The graphs show how the profiles change with different Froude numbers, illustrating the impact on the system’s behavior.

Navigate back to Figure 13..

## Figure 14. Long description

The image contains four sets of graphs, each with five subplots representing different Froude numbers. The graphs depict profiles of turbulent kinetic energy (TKE), dissipation rate, scalar variance, and scalar flux. Each set of graphs shows how these profiles evolve over time, with lighter lines representing earlier times and darker lines representing later times. The x-axes of the graphs use a logarithmic scale for TKE and dissipation rate. Blue dashed lines are present in each subplot, likely indicating a specific reference level or threshold. The graphs illustrate the changes in these parameters as the Froude number decreases from left to right.

Navigate back to Figure 14..

## Figure 15. Long description

A line graph with four data lines representing different Froude numbers (F r equals 14, 7, 3.5, and 1.25) plotted against the turbulent Froude number (F r t) on the x-axis and z on the y-axis. The x-axis ranges from 10 to the power of negative 2 to 10 to the power of 2, while the y-axis ranges from negative 2.5 to negative 1. The black vertical line marks a specific value on the x-axis, and horizontal dashed lines indicate where this value is reached for each simulation. The data lines show how z varies with F r t for each Froude number. All values are approximated.

Navigate back to Figure 15..

## Figure 16. Long description

The image contains six graphs arranged in two rows and three columns. The top row (a) shows outer scaling, while the bottom row (b) shows inner scaling. Each column represents different variables: kinetic energy, dissipation rate, and length scale. The graphs use various colors to represent different Froude numbers. In the outer scaling graphs, dashed grey lines indicate empirical fits, showing the breakdown of scaling due to buoyancy effects. Coloured circles and crosses mark specific points where certain conditions are met. The inner scaling graphs include vertical dashed grey lines marking where curves are within a half-standard deviation of each other. The graphs illustrate how these variables change with respect to normalized height.

Navigate back to Figure 16..

## Figure 17. Long description

A line graph with three data lines representing the relative location of z1 to the density interface, zt, zI, and zb, as a function of the Froude number. The x-axis represents the Froude number ranging from 1.25 to 14.00. The y-axis represents the normalized distance (z1 − zt)/δI, (z1 − zI)/δI, and (z1 − zb)/δI, ranging from −1 to 2. The blue line with circle markers shows (z1 − zt)/δI, the orange line with x markers shows (z1 − zI)/δI, and the yellow line with triangle markers shows (z1 − zb)/δI. All values are approximated.

Navigate back to Figure 17..

## Figure 18. Long description

A vertical dot plot displays data points representing outer, inner, and overlap regions at different depths for four cases. The x-axis represents different cases with varying Froude numbers (Fr = 14, Fr = 7, Fr = 3.5, Fr = 1.25), while the y-axis represents depth (z) ranging from −1.00 to −2.50. Each case is color-coded: orange for Fr = 14, yellow for Fr = 7, purple for Fr = 3.5, and green for Fr = 1.25. The plot uses circles to denote the outer region, squares for the inner region, and a combination of both for the overlap region. The data points are connected by dashed lines corresponding to their respective regions. The outer region data points are higher on the y-axis, while the inner region data points are lower. The overlap region data points are positioned between the outer and inner regions. The plot highlights the distribution and interaction of these regions across different depths and cases.

Navigate back to Figure 18..

## Figure 19. Long description

The image contains two line graphs. The first graph on the left shows the outer scaling with the x-axis labeled as z prime and the y-axis labeled as N squared. Multiple colored lines represent different data sets, with an open red circle marking a specific location for SM123. The second graph on the right displays normalized curves with the x-axis labeled as (z prime minus z zero) divided by l zero and the y-axis labeled as N squared divided by N zero squared. The spread of normalized curves at a specific location is 25.0 percent relative to the mean value at that location. The colored lines in both graphs represent different data sets.

Navigate back to Figure 19..

## Figure 20. Long description

Two line graphs compare isotropy factors for stratified simulations with different Froude numbers. The first graph (a) shows isotropy factors with the vertical axis labeled ‘z’ and the second graph (b) shows the same data with the vertical axis shifted to the forcing layer edge and normalized by the depth. Open circles mark the location where a specific condition is met, and closed circles in graph (a) indicate where another condition is met. The graphs use different colors to represent various Froude numbers: blue for infinity, orange for 14, yellow for 7, purple for 3.5, and green for 1.25. The lines and circles illustrate how the isotropy factor varies with depth and Froude number.

Navigate back to Figure 20..

## Figure 21. Long description

The image contains four separate graphs labeled (a), (b), (c), and (d), each depicting Reynolds stress anisotropy for different Froude numbers. The x-axis represents the normalized depth, while the y-axis represents the normalized height. Each graph includes multiple lines representing different components of Reynolds stress anisotropy. The blue line represents b_hh, the orange line represents b_33, the yellow dashed line represents b_12, the purple dashed line represents b_13, and the green dashed line represents b_23. The horizontal black lines mark the location where the normalized depth equals negative 0.5. Graph (a) shows results for Fr = 14, graph (b) for Fr = 7, graph (c) for Fr = 3.5, and graph (d) for Fr = 1.25. The lines intersect and diverge at various points, indicating the relationship between different components of Reynolds stress anisotropy at different depths and Froude numbers.

Navigate back to Figure 21..

## Figure 22. Long description

The image contains two line graphs labeled (a) and (b). Graph (a) shows the evolution of interface location, with dashed lines representing medium resolution cases and solid lines representing fine resolution runs. Graph (b) illustrates the evolution of two variables, with solid lines for one variable and dashdot lines for another. Black dotted lines approximate the linear portion of one of the variables. The time axis in graph (b) is zoomed in on the high-resolution time record. Time is normalized by the eddy turnover time in the forcing region. The graphs compare different resolutions and highlight the linear portion of the data.

Navigate back to Figure 22..

## Figure 23. Long description

The image contains two scatter plots side by side. The left plot (a) shows the ratio of velocity scales, with the x-axis labeled as ‘Ri_h = ΔbL_h/u_h^2^’ and the y-axis labeled as ‘w_s/u_h’. The right plot (b) displays length-scale ratios, with the x-axis labeled as ‘Ri_h = ΔbL_h/u_h^2^’ and the y-axis labeled as ‘L_E/L_h’. Both plots feature data points represented by black circles. The right plot includes a trend line labeled ‘Ri_h^(-1/2)^’. The x-axis values range from 0 to 400 in plot (a) and from 10 to 1000 in plot (b). The y-axis values range from 0 to 2 in plot (a) and from 0.1 to 10 in plot (b). All values are approximated.

Navigate back to Figure 23..

## Figure 24. Long description

A scatter plot showing entrainment rate versus Richardson number. The plot features blue circles representing the RMS horizontal velocity and integral scale in the homogeneous fluid, measured at the depth of the density interface. Red triangles represent the RMS vertical velocity and Ellison length scale measured where the buoyancy frequency is in the stratified fluid. The x-axis represents the Richardson number on a logarithmic scale, and the y-axis represents the entrainment rate on a logarithmic scale. Three trend lines are shown: a solid line indicating a power law proportional to Ri inverse, a dashed line indicating a power law proportional to Ri inverse squared, and a dash-dot line indicating a power law proportional to Ri inverse three-halves. The data points show a general trend of decreasing entrainment rate with increasing Richardson number.

Navigate back to Figure 24..

## Figure 25. Long description

The line graph illustrates the time evolution of the interface thickness for various Froude numbers. The x-axis represents time in units of t epsilon sub f divided by k sub f, ranging from 0 to 600. The y-axis represents the interface thickness delta sub I, ranging from 0 to 3. The graph includes five data lines, each corresponding to a different Froude number: Fr equals infinity, Fr equals 14, Fr equals 7, Fr equals 3.5, and Fr equals 1.25. The blue dashed line for Fr equals infinity shows a steady increase in interface thickness over time. The orange dashed line for Fr equals 14 shows a moderate increase, while the yellow dashed line for Fr equals 7 shows a smaller increase. The purple and green dashed lines for Fr equals 3.5 and Fr equals 1.25, respectively, show relatively stable interface thickness with minor fluctuations. All values are approximated.

Navigate back to Figure 25..

## Figure 26. Long description

A line graph presents the relationship between interface thickness and horizontal Froude number. The x-axis represents the horizontal Froude number on a logarithmic scale ranging from 10^-2^ to 10^0^. The y-axis represents the normalized interface thickness on a logarithmic scale ranging from 10^-1^ to 10^1^. The graph includes two data series: blue circles and grey triangles. The blue circles represent data evaluated at a specific location, while the grey triangles represent data evaluated at another location and have been multiplied by two for clear presentation. Solid lines indicate time-averaged interface thickness over a fine-resolution time record. The blue circles follow a trend line proportional to the horizontal Froude number raised to the power of 2/3, while the grey triangles follow a trend line proportional to the horizontal Froude number raised to the power of 3/4. All values are approximated.

Navigate back to Figure 26..

## Figure 27. Long description

The image contains two line graphs side by side. The left graph shows TKE against z for two different domain sizes, Lx equals 6 and Lx equals 9. The right graph shows TKE against z for three different zb values, zb equals negative 4, zb equals negative 5.0312, and zb equals negative 6.0625. Both graphs illustrate the precipitous drop of TKE marking the edge of the sponge layer. The x-axis represents TKE on a logarithmic scale, and the y-axis represents z. The lines in each graph represent different conditions, with color-coded lines for clarity. The left graph uses blue for Lx equals 6 and red for Lx equals 9. The right graph uses blue for zb equals negative 4, red for zb equals negative 5.0312, and yellow for zb equals negative 6.0625. All values are approximated.

Navigate back to Figure 27..

## Figure 28. Long description

The image contains four line graphs labeled (a) through (d), each depicting different aspects of a grid convergence study. Graph (a) shows turbulence kinetic energy, graph (b) shows dissipation (viscous + SGS), graph (c) shows transport, and graph (d) shows enstrophy. Each graph features four lines representing different values of n, ranging from 0 to 3. The x-axes of the graphs use a logarithmic scale, while the y-axes measure the respective variables. The lines converge as the grid resolution increases, indicating the study’s focus on how these variables change with grid refinement. All values are approximated.

Navigate back to Figure 28..

## Figure 29. Long description

The image contains six line graphs arranged in a 2x3 grid. Each graph plots a different variable against the vertical axis z. The variables include turbulence kinetic energy (TKE), TKE dissipation rate, temperature variance, temperature flux, temperature variance destruction, and temperature flux destruction. Each graph features three lines representing different values of n (n = 1, n = 2, n = 3). The x-axes of the graphs use logarithmic scales to display the range of values for each variable. The lines show how these variables change with respect to the vertical axis z. The graphs illustrate the relationships and trends between these variables in an unstratified case.

Navigate back to Figure 29..

## Figure 30. Long description

A line graph displays Ozmidov scale resolution with four distinct data lines representing different Froude numbers. The x-axis is labeled with the ratio of delta x to L O, ranging from 10 to the power of negative 4 to 10 to the power of 1. The y-axis is labeled with z values ranging from negative 3 to 0. The data lines are color-coded: orange for Fr equals 14, yellow for Fr equals 7, purple for Fr equals 3.5, and green for Fr equals 1.25. The horizontal black line marks the initial density interface location, and the vertical black line indicates where large eddy simulation should strive to be below to accurately measure mixing. All values are approximated.

Navigate back to Figure 30..

## Figure 31. Long description

The left side of the image displays a heat map of temperature contours, with blue representing lower temperatures and red indicating higher temperatures. The right side shows a scalar-bounding mask, where red areas indicate where the model is active and white areas show where the standard SGS model is used. The temperature contours reveal a gradient from blue at the bottom to red at the top, suggesting a transition from cooler to warmer regions. The scalar-bounding mask highlights specific regions of activity within the model.

Navigate back to Figure 31..

## Figure 32. Long description

The image contains three graphs showing the TKE profiles. The first graph (a) displays unscaled or raw data with different cases represented by various colors. The legend indicates the parameters for each case. The second graph (b) shows scaled data referenced to a specific value determined from fitting a straight line to the profiles. The third graph (c) is similar to the second but uses a different method to determine the scaling value. Black lines in the scaled TKE plots represent power laws corresponding to where the exponent is reported on the figures. The x-axes and y-axes vary across the graphs to show different aspects of the data.

Navigate back to Figure 32..

## Figure 33. Long description

The image contains three separate graphs. The first graph shows profiles of the turbulence length scale and the linear fits used in determining virtual origins. The x-axis represents z prime, and the y-axis represents k z prime squared over epsilon. The second graph displays profiles of one over u r m s and the linear fits used in determining virtual origins. The x-axis represents z prime, and the y-axis represents one over u r m s. The third graph illustrates the variation of z zero with forcing layer Reynolds number. The x-axis represents R e f, and the y-axis represents z zero. Different colored lines and markers represent various conditions or datasets.

Navigate back to Figure 33..
